# An aPKC-Exocyst Complex Controls Paxillin Phosphorylation and Migration through Localised JNK1 Activation

**DOI:** 10.1371/journal.pbio.1000235

**Published:** 2009-11-03

**Authors:** Carine Rosse, Etienne Formstecher, Katrina Boeckeler, Yingming Zhao, Joachim Kremerskothen, Michael D. White, Jacques H. Camonis, Peter J. Parker

**Affiliations:** 1 Protein Phosphorylation Laboratory, Cancer Research UK London Research Institute, London, United Kingdom; 2 Hybrigenics SA, Paris, France; 3 Biochemistry, University of Texas Southwestern Medical Center, Dallas, Texas, United States of America; 4 Department of Molecular Nephrology, University Clinic Muenster, Muenster, Germany; 5 Department of Cell Biology, University of Texas Southwestern Medical Center, Dallas, Texas, United States of America; 6 Institut Curie, Transduction Networks Analysis Group, Paris, France; 7 Division of Cancer Studies, King's College School of Medicine, London, United Kingdom; National Institute of Dental and Craniofacial Research, United States of America

## Abstract

Atypical protein kinase C (aPKC) isoforms have been implicated in cell polarisation and migration through association with Cdc42 and Par6. In distinct migratory models, the Exocyst complex has been shown to be involved in secretory events and migration. By RNA interference (RNAi) we show that the polarised delivery of the Exocyst to the leading edge of migrating NRK cells is dependent upon aPKCs. Reciprocally we demonstrate that aPKC localisation at the leading edge is dependent upon the Exocyst. The basis of this inter-dependence derives from two-hybrid, mass spectrometry, and co-immunoprecipitation studies, which demonstrate the existence of an aPKC–Exocyst interaction mediated by Kibra. Using RNAi and small molecule inhibitors, the aPKCs, Kibra, and the Exocyst are shown to be required for NRK cell migration and it is further demonstrated that they are necessary for the localized activation of JNK at the leading edge. The migration associated control of JNK by aPKCs determines JNK phosphorylation of the plasma membrane substrate Paxillin, but not the phosphorylation of the nuclear JNK substrate, c-jun. This plasma membrane localized JNK cascade serves to control the stability of focal adhesion complexes, regulating migration. The study integrates the polarising behaviour of aPKCs with the pro-migratory properties of the Exocyst complex, defining a higher order complex associated with the localised activation of JNK at the leading edge of migrating cells that determines migration rate.

## Abstract 2

The exocyst/aPKC complex controls the spatiotemporal activation of the kinases JNK and ERK at the leading edge of migrating cells and thereby controls the dynamic behaviour of the adhesion protein paxillin during cell migration.

## Author Summary

Cell migration is an essential process in multicellular organisms during such events as embryonic development, the immune response, and wound healing. Cell migration is also instrumental in the development of pathologies such as cancer cell invasion of healthy tissues. To make cells move, key molecules must be engaged in a coordinated manner; understanding which molecules, and how and when they work (for example, under physiological versus pathological conditions) will impact on new therapies designed to suppress abnormal migration. Migrating cells must coordinate two key processes: extension of the front or ‘leading’ edge of the cell and retraction of the back edge. Both processes require the turnover of protein assemblies known as focal adhesion complexes. In this paper we show that two different groups of regulators of migration – aPKC, a protein kinase, and exocyst, a complex of proteins also known to be required for exocytosis – interact physically via the scaffold protein kibra. All these components are required for efficient cell migration and all are enriched at the leading edge of moving cells, in a mutually dependent manner. At the leading edge, these components control the local activation of two additional protein kinases, ERK and JNK. The activation of ERK and JNK at the front of migrating cells in turn controls the phosphorylation of paxillin, a component of focal adhesions. Phosphorylation of paxillin is associated with the presence of more dynamic focal adhesions. Our data thus indicate that an aPKC-kibra-exocyst complex plays a crucial role in delivering local stimulatory signals to the leading edge of migrating cells.

## Introduction

Migration of cells is critical to the development and the normal physiology of organisms; it also plays a more sinister role in the dissemination of cancer towards metastatic disease, a process typically associated with poor prognosis. The process of migration involves a combination of cellular functions including those of altered attachment to surrounding contacts (cells or matrix), protrusion of a leading edge, polarisation in the creation or recognition of that leading edge, and mechanical movement [1]. Understanding the details of these processes represents an important objective in defining the collection of candidate targets that may offer new opportunities in restricting disease spread.

The atypical PKC isoforms (aPKCζ and aPKCι) comprise a branch of the serine/threonine protein kinase PKC superfamily with regulatory properties that distinguish them from the more typical diacylglycerol-regulated isoforms [2]. These kinases can be activated by acidic phospholipids such as the polyphosphoinositides [3], however specificity appears to be driven by activation through Par6/cdc42 [4]. Indeed, interactions with the Par6/Par3 complex have implicated aPKC isoforms in a number of polarity [5] and more recently migratory models [6]. In migrating astrocytes, the activation of aPKC leads to phosphorylation and inactivation of GSK-3β, which causes the adenomatous polyposis coli (APC) tumor suppressor protein to associate with microtubule plus ends at the leading edge [7]. The Par6-PKCζ complex also regulates the spatially localized association of Dlg1 and APC to control cell polarization [8]. PKCζ is required for epidermal growth factor-induced chemotaxis of human breast cancer cells [9], while PKCι has been shown to promote nicotine-induced migration and invasion of cancer cells via phosphorylation of m- and μ-calpains [10].

The Exocyst was first identified as a complex required for exocytosis in *Saccharomyces cerevisiae* [11]. In mammals, the Exocyst comprises a complex of eight proteins, which facilitates regulated exocytosis to regions of membrane activity [12],[13],[14],[15]. Recently it has been shown that a Ral-Exocyst pathway is involved in cell migration, with RalB activation leading to Exocyst assembly and recruitment to the leading edge [16].

It is anticipated that the various complexes and pathways involved in polarized migration may be co-regulated/coordinated and that elucidation of these relationships will lead to a more integrated understanding of migratory behaviour. The present study was stimulated by the finding that the scaffold protein Kibra, previously shown to interact with aPKCζ [17], was a binding partner of the Exocyst (see below). This has led to the specific hypothesis that there is a molecular and functional interaction between the Par/CDC42/PKCζ/ι pathway and the Ral/Exocyst pro-migratory pathways. We demonstrate that indeed there is a mutual dependence of aPKC and Exocyst in their behaviour in migratory cells and this is associated with their mutually dependent regulation of the delivery of signals to the leading edge of migrating cells. We identify key regulatory processes under the control of these local aPKC/Exocyst-dependent signals and go on to demonstrate that the regulation of focal adhesion stability represents a critical migratory output of the aPKC/Exocyst pathway.

## Results

### Atypical PKCs Are Necessary for NRK Cell Migration

**Figure 1:**
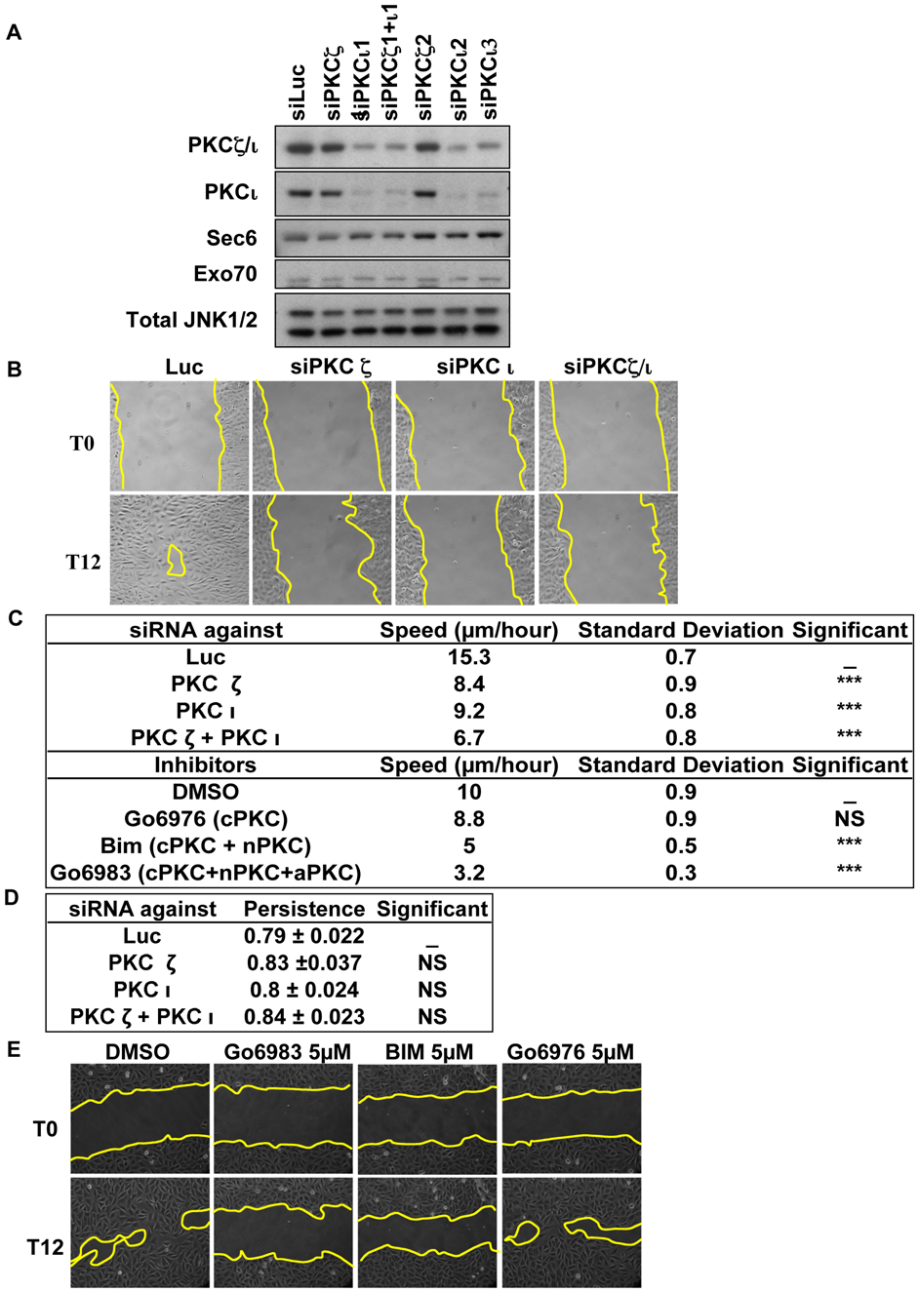
aPKCs are required for cell migration. (A) siRNA specificity and efficiency in NRK cells. For the siRNA experiments, NRK cells were transfected with the indicated siRNAs. After 72 h, whole-cell extracts were analyzed by immunoblotting with the indicated antibodies. (B) Depletion of PKCζ, PKCι, or depletion of both inhibits wound healing. Confluent monolayers of NRK cells transfected with the indicated siRNA were wounded 60 h post-transfection (T0), and healing was followed over time. T12 refers to the time 12 h post-wounding. The panels shown are representative of at least three independent experiments. (C) Quantitation of migration speed and the effects of aPKC knock-down or inhibitors is summarized in the panel. (D) Quantitation of migration persistence and the effects of aPKC knock-down are summarized in the panel. (E) NRK cells were pretreated for 6 h with Gö6976 (5 µM; cPKC inhibition), BIM (5 µM; c/nPKC inhibition), or Gö6983 (5 µM; c/n/aPKC inhibition) and then the confluent monolayers were wounded. The healing was followed over 12 h. TO and T12 refer, respectively, to the times 0 h and 12 h post-wounding. The panels shown are representative at least of three independent experiments.

A cooperative functional relationship between the Exocyst complex and aPKC in cell migration should be reflected in a shared requirement in a model system. The Exocyst has been shown previously to play an essential role in NRK cell migration. So to determine the requirements for PKCι and PKCζ in NRK cell migration, two independent siRNAs for each protein were employed to knock-down expression (Figure 1A). Depletion of either PKCι or PKCζ resulted in a cell migration defect as assessed in a monolayer wound healing model (Figure 1B) as well as in a Transwell migration assay (unpublished data). The speed of NRK cell migration in the wound assay is 15.3 µm/h. Depletion of either aPKC reduces this by ∼40%, whilst depletion of both reduces migration further to ∼6 µm/h (Figure 1C).

To distinguish between a non-catalytic, scaffold-only requirement for aPKC and a catalytic activity requirement, PKC inhibitors were employed. Selected combinations of these inhibitors can be exploited to provide circumstantial evidence on the requirements of PKC isoforms based upon their relative potency such that an aPKC activity involvement would be sensitive to the pan-PKC inhibitor Gö6983 (cPKC, nPKC, aPKC) while showing little sensitivity to BIMI (cPKC, nPKC) or Gö6976 (cPKC). Gö6983 had a profound effect on cell migration, while the cPKC inhibitor Gö6976 had no effect (Figure 1C and 1D). BIMI had a weaker effect than the Gö6983 on migration (Figure 1C and 1D). Although these inhibitors are not entirely PKC-specific, in combination with the siRNA data it can be concluded that aPKCs are required for efficient NRK cell migration, providing a model in which to probe an aPKC-Exocyst relationship in migration.

### aPKCs and the Exocyst Complex Are Recruited to the Leading Edge of Migrating Cells in a Mutually Dependent Manner

**Figure 2:**
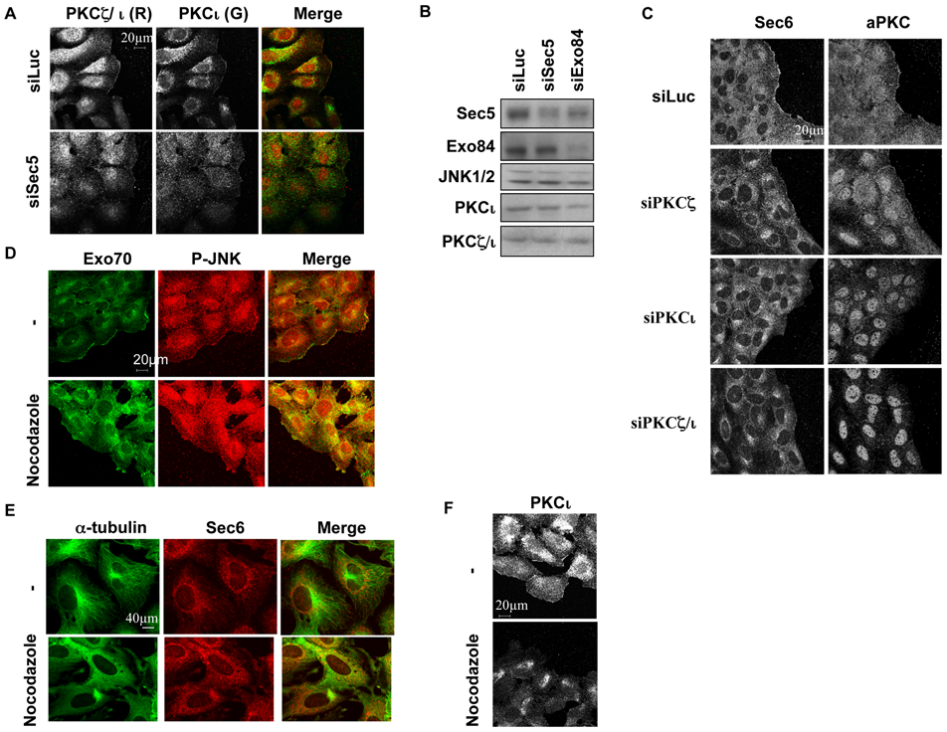
Mutual, microtubule dependent control of aPKC and the exocyst at the leading edge. (A) Monolayers of NRK cells were wounded 60 h post-transfection with the indicated siRNAs (10 nM). Cells were fixed 6 h after wounding, and the indicated proteins were detected with specific antibodies. PKCζ and PKCι are recruited at the leading edge and these recruitments are dependent on the presence of Sec5. (B) siRNA specificity and efficiency in NRK cells. For the siRNA experiments, NRK cells were transfected with the indicated siRNAs. After 72 h, whole-cell extracts were analyzed by immunoblotting with the indicated antibodies. (C) Monolayers of NRK cells were wounded 60 h post-transfection with the indicated siRNAs (10 nM). Cells were fixed 6 h after wounding with paraformaldhehyde, permeabilized by Triton×100 1%, and then immunostained for Sec6 or aPKC. (D–F) Monolayers of NRK cells were wounded, and 5 h post-wounding, cells were treated for 1 h with nocodazole (10 µM). Cells were then fixed with paraformaldhehyde, and Exo70, P-JNK1/2, and PKCι were detected with specific antibodies. (E) Cells were fixed with methanol and stained for Sec6 and Fitc-tubulin.

To assess a connection between aPKC isoforms and the Exocyst, we sought to determine their distribution in migrating cells. Examination of the location of aPKCs (combined PKCι and PKCζ) demonstrated that aPKC was localised at the leading edge of migrating NRK cells (Figure 2A). By contrast, in confluent cells, aPKCs are mainly cytosolic and sometimes partially at cell-cell contacts (unpublished data). A monoclonal antibody specific for PKCι confirmed its presence at the leading edge as well as within a perinuclear compartment; PKCζ specific reagents were found to cross-react with recombinant PKCι precluding PKCζ-specific immunostaining (Dr. M. Linch and PJP unpublished results). The Exocyst (here visualized by the subunits Sec6 and Exo70) is also in part localized at the leading edge of migrating cells and the pattern of aPKC distribution matches that observed for the Exocyst complex. The localisation of aPKC and the Exocyst at the leading edge of migrating cells is not simply a function of membrane ruffling. In subconfluent monolayers extensive membrane ruffling is observed without enrichment of aPKC or the Exocyst (Figure S1), while the ruffles at the leading edge of migrating cells are enriched with aPKC and the Exocyst.

**Figure 3:**
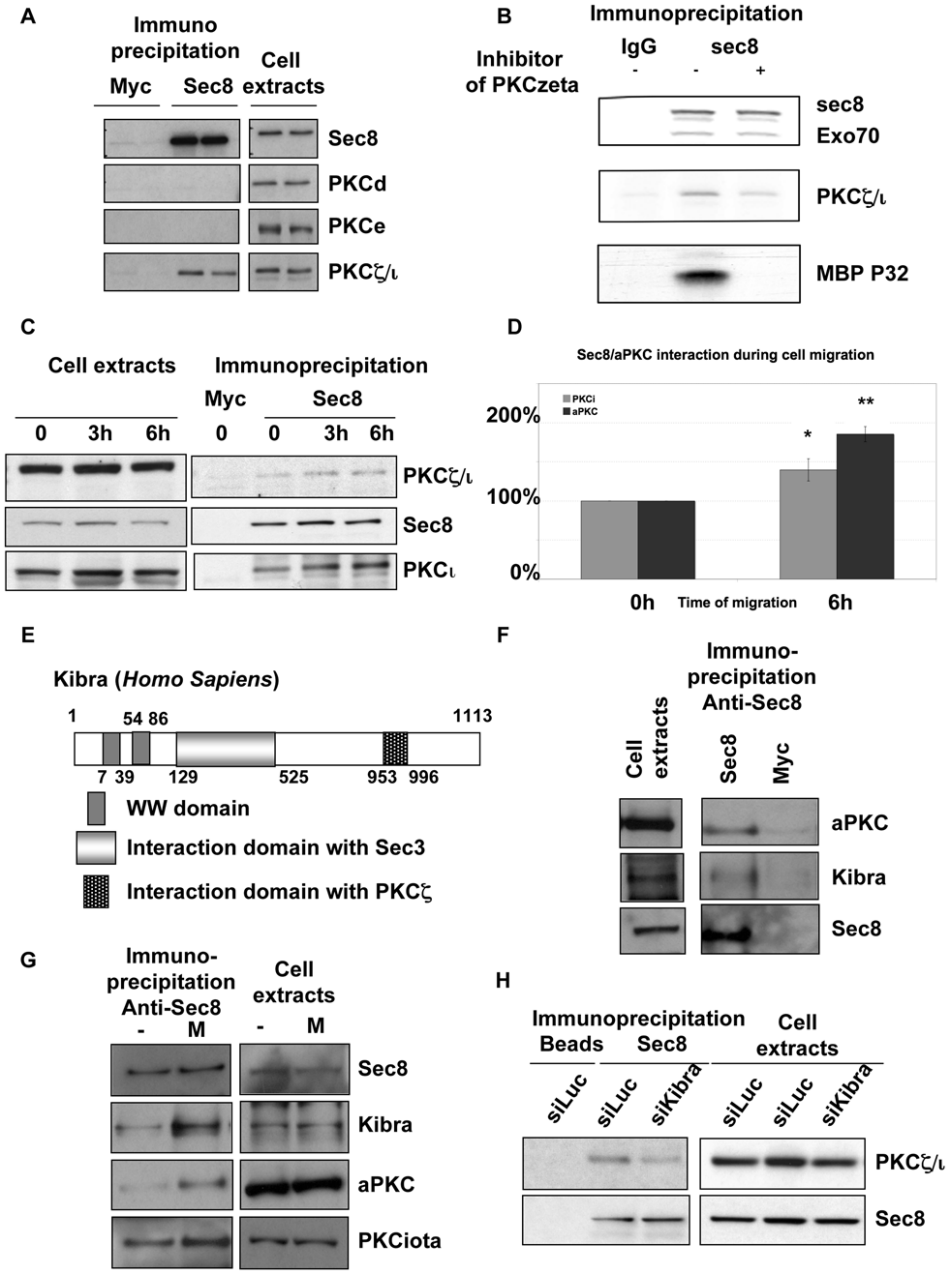
The Exocyst interacts with active aPKC via kibra in a migration-dependent manner. (A) Protein extracts from NRK cells were immunoprecipitated with anti-Sec8 or anti-myc antibodies and the immunoprecipitates were analysed for the presence of PKCε, PKCδ, and PKCζ/ι by Western blotting. (B) The Exocyst was recovered by immunoprecipitation of Sec8 and associated kinase activity was assessed in the presence of ^32^P-ATP and an exogenous PKC substrate, MBP, either in the presence or absence of an aPKC inhibitor. (C) As previously described [16], a monolayer of confluent NRK cells was extensively scratched to maximize the number of “free edges” where cell-cell contacts were released. Sec8 was immunoprecipitated and PKCι and PKCζ/ι were detected by Western blot. The loading of the cell extracts represents 10% of the input for immunoprecipitation. (D) Quantitation of three experiments as described in (C). (E) Depiction of the domains of interaction between Kibra/Sec3 and also between Kibra/PKCζ. (F) A monolayer of confluent NRK cells overexpressing Myc-Kibra was extensively scratched to maximize the number of “free edges” where cell-cell contacts were released. Myc-Kibra was immunoprecipitated and Sec8 was detected by Western blot. (G) NRK cells were transfected with a siRNA against Kibra or an siRNA control as indicated. Seventy-two h after transfection, the monolayers of confluent NRK cells were extensively scratched to maximize the number of “free edges” where cell-cell contacts were released. Sec8 was subsequently immunoprecipitated, and associated aPKCs were detected by Western blot. (H) Immunoprecipitates of Sec8 were performed from cells treated with an siRNA directed at Kibra or with a luciferase siRNA control as indicated. Immunocomplexes were then probed for atypical PKC and Sec8 itself.

To determine whether the Exocyst was responsible for aPKC accumulation at the leading edge, siRNA to Sec5 was employed [16]. Knock-down of Sec5 (Figure 2A) prevented aPKC localisation at the leading edge without disturbing the total amount of aPKC in the cells (Figure 2B). Knock-down of another component of the Exocyst complex, Exo84, also prevented aPKC localisation at the leading edge (see below, Figure 3C). When one of the components of Exocyst complex (either Sec5 or Exo84) is depleted, more than 50% of the cells have a total absence of PKCι at the leading edge. Consistent with this, migration was inhibited on knock-down of Sec5 and Exo84 by 60%. Reciprocally it was found that siRNA to aPKCs (Figure 2C and Figure S2A, S2C) or treatment with the inhibitor Gö6983 (unpublished data) suppressed Exo70 (for more than 60% of the cells) and Sec6 accumulation at the leading edge. This disruption of localisation is not due to a modification of the protein levels of Exo70 and Sec6 (Figure 1A). The recruitment of aPKC and Exo70 at the leading edge were quantified (Figure S2A, S2B, S2C). We also evaluated the specificity of the staining as well as the effect on the knock-down of the Exocyst (Sec5 or Exo84) on the presence of aPKC at the leading edge and reciprocally the effect on the knock-down of aPKC on the presence of Exo70 at the leading edge (Figure S2A, S2B). Notably, depletion of either PKCι or PKCζ partially inhibited the “tubularisation” of the Sec6 compartment seen in motile cells on methanol fixation (Figure S3C), suggesting that aPKC is required for the localization of Sec6 on microtubules in response to cell migratory cues. Depletion of PKCι or PKCζ or Sec5 did not dramatically affect the stability of the microtubules (unpublished data). To control for the possibility of global disruptive effects of aPKC on vesicle-staining patterns in migrating cells, β-COP proteins were monitored. The depletion of aPKC modified the localization of Sec6 but not β-COP, indicating the specificity of the aPKC effect (Figure S3C).

### The Recruitment of aPKCs and the Exocyst Complex at the Leading Edge Are Dependent on Microtubules

The Exocyst associates with microtubules [18],[19] providing a potential basis for Exocyst and aPKC movement. As predicted, depolymerisation of the microtubule network with nocodazole was found to block the accumulation of Exo70 at the leading edge (Figure 2D). Using methanol fixation, Sec6 is detected on tubular structures and these “tubules” of Sec6 are also dependent on the stability of the microtubules (Figure 2E). Because aPKCs were described to control MTOC orientation via the Dynein-Dynactin complex [20] and the recruitment of the Exocyst complex is dependent on the microtubule network, it is possible the effect of the depletion of aPKC on the recruitment of the Exocyst at the front of the cells is due to an indirect effect of aPKC on motor proteins. However, the presence of dominant negative CDC42, which is involved in aPKC effects on polarity, did not disturb the localization of Exo70 at the leading edge (Figure S3D). As predicted from the observations above, nocodazole treatment also blocks aPKC delivery to the leading edge (Figure 2F). The specificity of nocodazole treatment is reflected in the finding that treatment increases actin stress fibres showing that cells retained microfilament structures (unpublished data).

### A Regulated Interaction of aPKCs and the Exocyst Complex in Cells

To investigate the basis of the mutual aPKC-Exocyst localisation relationship, assessment was made of the possible association of aPKC and the Exocyst in NRK cells. Antibodies to the Exocyst subunit Sec8 efficiently immunoprecipitated the native complex; probing this immunopurified complex for the presence of aPKC showed that aPKC was also associated. By contrast the related PKCε and PKCδ, which are also expressed in NRK cells, were not recovered in association with the immunopurified Exocyst complex (Figure 3A). Sec8 interacts with both PKCι and PKCζ, based on immunoprecipitation from NRK cells expressing PKCι or a myc tagged PKCζ construct (Figure S4A and S4B). Immunoprecipitation with anti-mycPKCζ or PKCι antibodies did not recover detectable Sec8 in the immunoprecipitate; it would seem that only a subfraction of PKCι or PKCζ is complexed to Sec8. Direct demonstration of the interaction in cells employing FRET approaches has not proved possible because the GFP-fusions of Exocyst subunits have been found not to enter into mature Exocyst complexes (unpublished data) in contrast to the findings in yeast [21].

Given the predicted requirement for activity in the migratory behaviour described above, it was important to determine whether the Exocyst associated aPKC was catalytically active. MBP was selectively phosphorylated in Sec8 immunoprecipitates and this phosphorylation was inhibited by a peptide inhibitor of aPKC (Figure 3B). The Sec8-associated aPKC thus appears to be active.

To assess if the interaction between aPKCs and the Exocyst is regulated, we examined activity and migratory requirements. Sec8 was immunoprecipitated following treatment with the inhibitor Gö6983. As shown in Figure S4C, the physical interaction between Sec8 and aPKC is not modulated in the absence of aPKC activity. To examine if cell migratory cues impact the stability of the interaction between the Exocyst and aPKC, a monolayer of confluent NRK cells was extensively scratched to maximize the number of “free edges” where cell-cell contacts are released. As shown in Figure 3C, cell migration promoted the interaction between aPKC and the Exocyst at 3 h and 6 h. The interaction with the Exocyst increased 1.5- to 2-fold (PKCι or both aPKCs) 6 h after monolayer wounding (Figure 3D). This increase of interaction during cell migration does not appear to be a non-specific stress response triggered by the multi-scratch assay, since no such influence is exerted by osmotic shock (unpublished data). These results sustain the idea that the function of these proteins requires their interaction during cell migration.

### aPKCs Interact with the Exocyst Complex Via Kibra, Which Is Also Required for Cell Migration

The Sec3 subunit of the Exocyst was used as a bait in a two-hybrid screen of a highly complex human placenta cDNA library (10 million independent clones). A total of 120 million interactions were screened (12 times library coverage) and four clones encoding human Kibra (NP_056053) were identified; Kibra is a known aPKC binding partner [17],[22]. Kibra's domain of interaction with Sec3 was defined by the smallest identified prey fragment and encompasses amino acids 129–526. This region has been found only in six screens amongst 935 screens performed against the same cDNA library, indicating that the Sec3-Kibra interaction is highly specific (Figure 3E). In the same screen with Sec3 as a bait, expected partners such as Sec5 (12 fragments, 4 different fusions; interacting domain is amino acids 96–252) and Sec8 (7 fragments, 2 different fusions; interacting domain is amino acids 28–167) were identified also. Confirmation of this Exocyst complex has come from independent studies as Kibra was identified as a partner of the Exocyst from an unbiased proteomic analysis of Exocyst-interacting proteins [23]. To determine the retention of this Kibra interaction in the context of the Exocyst complex, we submitted protein extracts from NRK cells expressing Myc-Kibra or Flag-Kibra constructs to immunoprecipitation with anti-Myc or Flag antibody and the immunoprecipitates were analyzed for the presence of Sec8 (a component of the complex but not Sec3 itself). Figure S5A and S5B show that ectopically expressed Myc-Kibra or Flag-Kibra co-immunoprecipitate with Sec8, whereas the beads alone or a myc antibody used as a negative control precipitated neither. For consistency, we tested if the interaction between Kibra and Exocyst (Sec8) and also between Kibra and aPKC are dependent on cell migration. As shown in Figure 3F, cell migration promoted the interaction between aPKC and Myc-Kibra and also that between Myc-Kibra and Sec8.

To ensure these observations were not a function of ectopic expression, we investigated the behaviour of the endogenous proteins. Endogenous Kibra was found to interact with Sec8 and aPKC, and this complex also increased during cell migration as observed above for the overexpressed Kibra (Figure 3F and 3G). This result sustains the idea that the function of these proteins requires their interaction during cell migration and is entirely consistent with the increased interaction between Sec8-aPKC during cell migration (Figure 3C).

**Figure 4:**
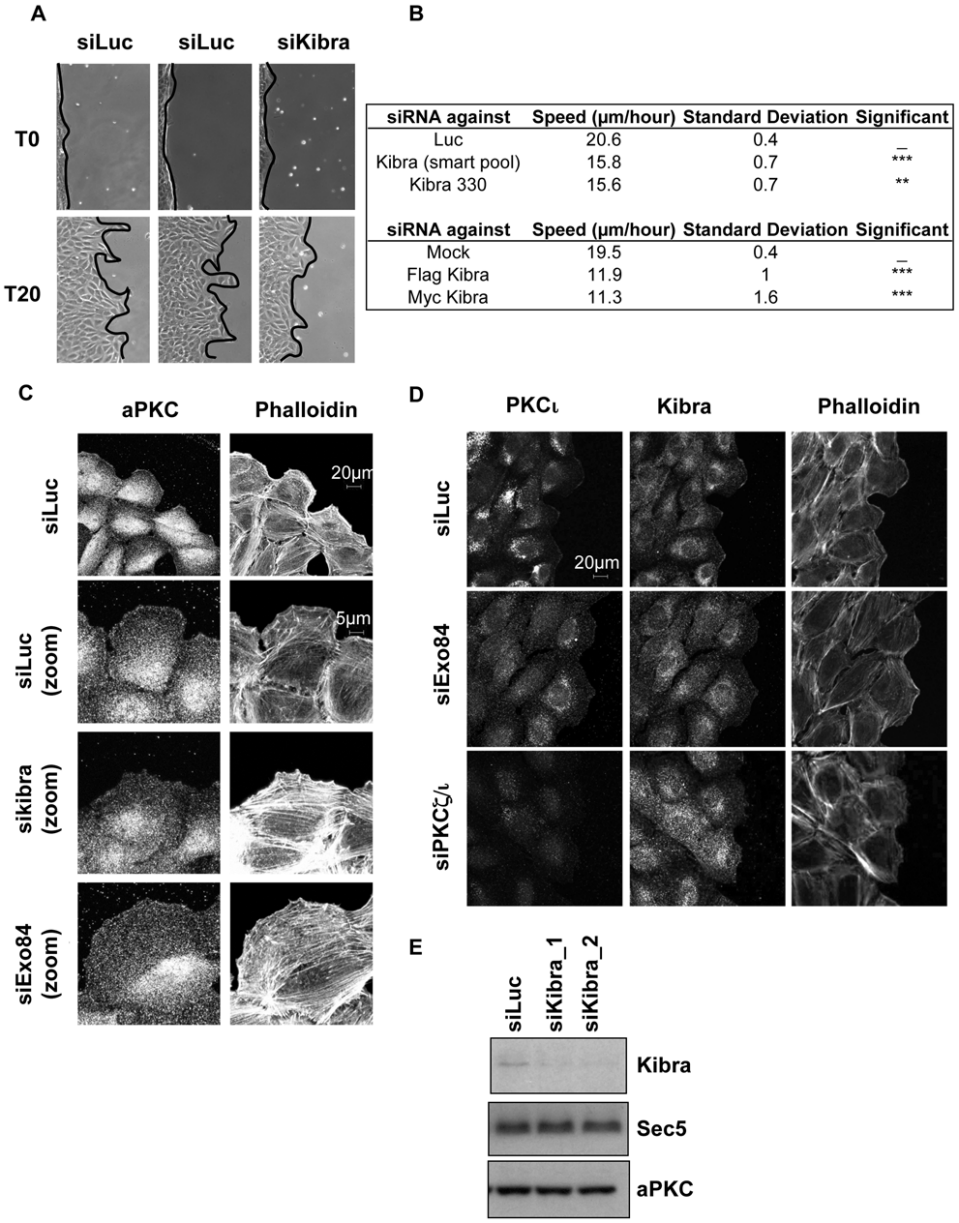
Kibra is required for cell migration and for the recruitment of aPKC at the leading edge. (A) Depletion of Kibra inhibits wound healing. Confluent monolayers of NRK cells transfected with the indicated siRNAs were wounded 60 h post-transfection (T0) and migration was followed over time. T20 refers to the time 20 h post-wounding. The panels shown are representative of at least of three independent experiments. Quantitation of migration and the effects of Kibra knock-down and overexpressed Kibra is summarized in panel (B). (C–D) Monolayers of NRK cells were wounded 60 h post-transfection with the indicated siRNAs (10 nM). Cells were fixed 6 h after wounding with paraformaldhehyde, permeabilized with 1% Triton×100, and then immunostained for PKCζ/ι, PKCι, Kibra, and actin (Phalloidin).

Given that the region of interaction between Kibra and Sec3 defined by two-hybrid resides within the amino acid sequence 129–526 and the region of Kibra that binds PKCζ encompasses amino acids 953–996, there is no apparent conflict for Kibra in binding both PKCζ and Sec3. To assess whether the endogenous aPKCs are associated with the endogenous Exocyst via Kibra, we submitted protein extracts from scratch wounded NRK cells, depleted or not of endogenous Kibra, to immunoprecipitation with anti-Sec8. The immunoprecipitates were analyzed for the presence of PKCζ/ι. The interaction between Sec8 and PKCζ/ι decreased on depletion of Kibra, demonstrating that Kibra contributes to complex formation (Figure 3G and Figure S5D). Based upon this requirement for Kibra, it was predicted that Kibra knock-down by siRNA would inhibit migration and this was found to be the case (Figure 4A, 4B, and 4E). The fact that there was consistently only a 25% decrease in cell migration in the absence of Kibra using different knock-down strategies suggests that an alternate protein(s) might also participate in the complex between the Exocyst and aPKC.

We sought to assess the relevance of the aPKC-Kibra interaction in the context of the localisation of aPKC in migrating cells. As previously shown in podocytes [24], endogenous Kibra was found to be recruited to the leading edge as well in NRK cells. Endogenous Kibra colocalised with aPKC in an Exocyst dependent manner (Figure 4D). The depletion of aPKC decreased the recruitment of Kibra at the leading edge (Figure 4C, 4D, and 4E) consistent with the lack of recruitment of the Exocyst under these conditions (see above). Interestingly, overexpression of Myc-Kibra or Flag-Kibra inhibits NRK cell migration (Figure 4B), consistent with interference in the bivalent interaction observed here. To address this point, examination of the location of aPKCs (combined PKCι and PKCζ) demonstrated that aPKC was colocalised at the leading edge of migrating NRK cells when Myc-Kibra is weakly overexpressed but the recruitment of aPKC at the leading edge is disturbed when Myc-Kibra is strongly overexpressed (Figure S5C). Finally, to determine whether Kibra is responsible for aPKC delivery to the leading edge, siRNA to Kibra was employed. Knock-down of Kibra inhibited aPKC localisation at the leading edge (Figure 4C, 4E).

### Atypical PKCs Control the Local Activation of JNK1 and ERK1/2 at the Leading Edge

**Figure 5:**
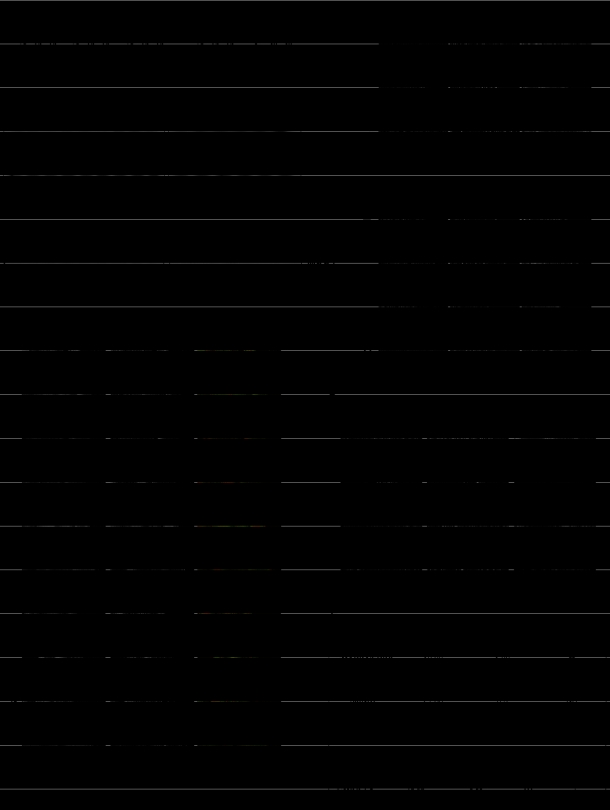
aPKC-dependent JNK1, ERK2, and MKK4 activation are required for NRK cell migration. (A) Monolayers of confluent NRK cells were extensively scratched to maximize the number of “free edges” where cell-cell contacts were released. Cells were treated with PKC inhibitors (Gö6976 or Gö6983 at 5 µM) for a total of 6 h, with extracts made at the indicated intervals post-wounding. Phospho-JNK1/2, total JNK1/2, phospho-MKK4, total MKK4, phospho MKK7, and total MKK7 were detected by Western blotting. (B,C,D) Monolayers of NRK cells were wounded 60 h post-transfection with the indicated siRNAs (10 nM). Cells were fixed 6 h after wounding with paraformaldhehyde, permeabilized with 1% Triton×100, and then immunostained for P-JNK, P-ERK, and actin (Phalloidin) as indicated in the individual panels. (B) Each image is the sum of the z-stack of confocal slices. (C–D) Each image is a zoom and one confocal slice. (E–F) Quantitation of migration speed and the effects of MKK4, MKK7, JNK1, JNK2, ERK1, and ERK2 knock-down is summarized in the panel. The controls monitoring the depletion of these proteins are presented in Figure S7.

The function of the leading edge aPKC-Kibra-Exocyst complex was assessed in relation to the activation of the JNK pathway, since there is evidence both for aPKC involvement in JNK control in other contexts (e.g. in response to TNF or IL1 [25],[26]), as well as for JNK involvement in migration [27],[28],[29],[30]. In response to wound healing (multiple scratch wounds), JNK1 and JNK2 were activated in a biphasic fashion (Figure 5A). The effect of acute inhibition of aPKC was assessed using Gö6983 and for comparison the non-aPKC directed inhibitors, Gö6976 (Figure 5A) and BIMI (unpublished data). The inhibition of atypical, novel, and classical PKCs suppressed the activation of JNK1 without affecting JNK2. By contrast the PKC inhibitors not directed at the aPKCs, Gö6976, and BIMI modestly increased JNK1 phosphorylation (Gö6976 decreases JNK2 activation while BIMI had no such effect, indicative of a non-PKC dependent effect). On depletion of JNK1 by siRNA, the P-JNK1 immunoreactivity was also decreased indicating that the doublet identified by western is JNK1 and a splice variant/modified form of JNK1 (Figure S7D). Inhibiting aPKC (Gö6983), but not non-aPKC family members (Gö6976 nor BIM1), also affected the activation of ERK1/2 during cell migration (Figure S4D, S4E). None of these PKC inhibitors influenced the p38 pathway response in migrating NRK cells, indicative of specific pathways wherein aPKC activity is required for JNK1 and ERK1/2 activation during migration.

**Figure 6:**
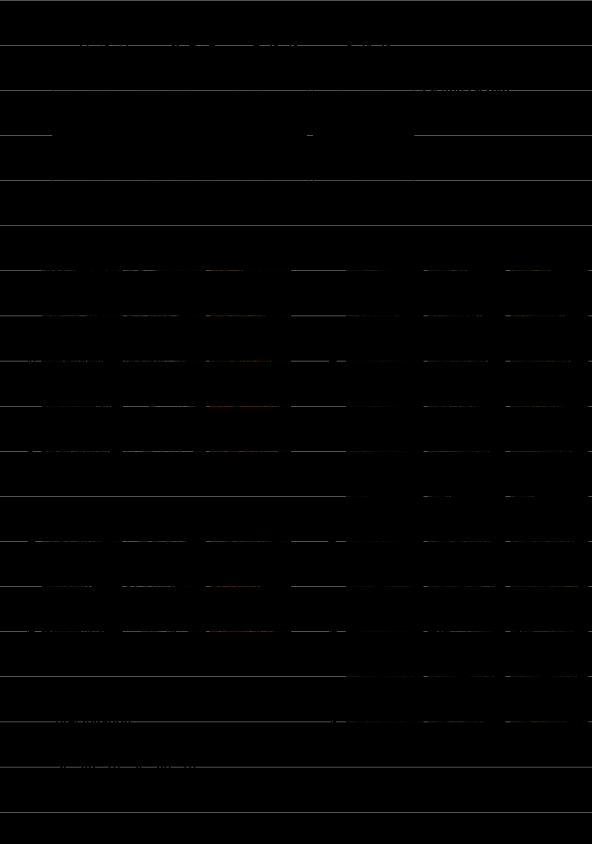
During cell migration aPKCs and the exocyst control the phosphorylation and the stability of paxillin at focal adhesions. (A) Monolayers of confluent NRK cells were extensively scratched to maximize the number of “free edges” where cell-cell contacts were released. Cells were treated with PKC inhibitors (Gö6976 or Gö6983 at 5 µM) for a total of 6 h, with extracts made at the indicated intervals post-wounding. Phospho-cJun (ser63), phospho-Paxillin (ser178), total cJun, and total Paxillin were detected. (B) Monolayers of NRK cells were wounded 60 h post-transfection with the indicated siRNAs (10 nM). Cells were fixed 6 h after wounding with paraformaldhehyde, permeabilized with 1% Triton×100, and then immunostained for actin (Phalloidin) and Paxillin as indicated in the individual panels. Each image is a projection of confocal slices. (C) Monolayers of NRK cells were wounded 60 h post-transfection with the indicated siRNAs (20 nM). Cells were fixed 6 h after wounding with paraformaldhehyde, permeabilized with 1% Triton×100, and then immunostained for P-JNK1/2 and Paxillin as indicated in the individual panels. Each image is a confocal slice. (D) A monolayer of confluent NRK cells was extensively scratched to maximize the number of “free edges” where cell-cell contacts were released. Sec8 was immunoprecipitated and Paxillin was detected by Western blot.

The effects of aPKC on JNK1 were reflected in the altered localised activation of JNK1/2 in migrating cells; under control conditions, JNK was found to accumulate in a phosphorylated state at the leading edge and this localised activation was lost on depletion of PKCζ, PKCι, Sec5, Exo84, or Kibra (Figure 5B–5D; see also Figure 6C, Figure S6A). Notably the depletion of these proteins did not influence the accumulation of active JNK in the nucleus, consistent with a lack of effect of Gö6983 on global JNK2 activation determined by Western. This Exocyst/Kibra/aPKC-dependence was also observed for the phosphorylation of ERK1/2 at the leading edge of migrating cells (Figure 5B–5D; see also Figure S6A). Quantitation of these responses is detailed in Figure S8. This confirms that there is a strong decrease of P-JNK and P-ERK at the leading edge without a significant disruption of the recruitment of the total ERK and JNK proteins at the leading edge (see below). It is noted that this demonstrates that this polarised leading edge in the migrating cells is still present even when aPKC and the Exocyst are non-functional. We confirmed the specificity of the phospho-specific antibody against P-ERK1/2 by using the MEK inhibitor U0126 and found that it suppressed the P-ERK staining (Figure S6B). We confirmed the specificity of the phospho-specific antibody against P-JNK1/2 by using a phospho-JNK (Thr183/Tyr185) blocking peptide and found that it suppressed the P-JNK staining (Figure S7F). Also, following depletion of JNK1 by siRNA, the staining of P-JNK at the leading edge decreased (Figure S7G). The controls monitoring the reduced protein expression associated with siRNA treatment is illustrated in Figure S7A and S7B (it is notable that Exo84 is reduced on Sec5 depletion suggesting that uncomplexed Exo84 may be subject to degradation).

This aPKC/Exocyst-dependent localised phosphorylation of MAPKinases (phosphorylated ERK1/2, as well as phosphorylated JNK) may be a consequence of their movement to the leading edge or of their activation at the leading edge. To distinguish between these two possibilities, the localisation of the kinases (phosphorylated and non-phosphorylated) was assessed in control cells or following aPKC, Kibra, Sec5, or Exo84 knock-down (protein depletion on knock-down is illustrated in Figure S7A and S7B). No effects on MAPkinase protein distribution were observed (Figure S6C, S6D, and S6E). Figure S6E shows that when the Exocyst/Kibra/aPKC is disrupted, P-ERK1/2 decreased at the leading edge whereas the non-phosphorylated form remained at the leading edge (note that the P-ERK1/2 staining appears a little different from that in Figure 5B–5D due to methanol fixation instead of paraformaldehyde fixation; the methanol fixation is better for the total ERK1/2 whereas the paraformaldehyde gave better staining for the P-ERK1/2). This result showed that cells retain a leading edge allowing the recruitment/retention of JNK and ERK proteins independent of the Exocyst and aPKC. Hence it is the localised activation that is critically under aPKC-Kibra-Exocyst control.

Analysis of the relevant upstream kinase(s) in the JNK pathway identified MKK4 and not MKK7 as showing increased phosphorylation after wounding (Figure 5A). Linking to aPKC function, wound-associated activation of MKK4 during cell migration is sensitive to the a/n/cPKC inhibitor Gö6983 (Figure 5A); Gö6976 (n/cPKC inhibitor) has no such effect (in fact it has the opposite effect to Gö6983, increasing the phosphorylation of MKK4). The constitutive phosphorylation of MKK7, the other JNK-kinase, was insensitive to Gö6983 (unpublished data).

### aPKCs Regulate JNK Phosophorylation of Its Plasma Membrane Substrate Paxillin but not of Its Nuclear Substrate, c-Jun

The above evidence is indicative of a localised MKK4-dependent activation of JNK1 during cell migration, requiring the localised action of the aPKC-Kibra-Exocyst complex. To assess the specificity of these plasma membrane effects in relation to other compartments, we compared the aPKC-dependency of the phosphorylation of nuclear (c-Jun) and plasma membrane (Paxillin) both substrates for JNK [31]. The aPKC inhibitor Gö6983 blocked the phosphorylation of Paxillin on serine 178 but not of c-jun serine 63 (Figure 6A). Furthermore, siRNA directed against aPKCs or Sec5 does not decrease phospho-cJun in the nucleus (Figure S6E). The lack of effect on Jun phosphorylation is consistent with the fact that phospho-JNK in the nucleus is not modified after depletion of aPKCs (see Figure 5B). Demonstrating that these events are integral to the observed migratory response, it was shown that the depletion of ERK2 but not ERK1, depletion of JNK1 but not JNK2, and depletion of MKK4 (and also MKK7) significantly decreased cell migration (Figure 5E and 5F) as did the JNK inhibitor, SP600125 (Figure S7H and S7I; the controls for depletion are included in Figure S7C and S7F).

To address the effect of the absence of aPKC and Exocyst on Paxillin in migrating NRK cells, cells were stained for Paxillin and Actin. More Paxillin patches at focal adhesion complexes and also more actin stress fibres appeared in the absence of aPKC and the Exocyst (Figure 6B; quantified in Figure S9). Figure 6C shows that P-JNK colocalised with Paxillin and if the aPKC/Exocyst pathway is disrupted, P-JNK at the leading edge is abolished whereas there is an increase of Paxillin patches. This increase of Paxillin patches was mirrored with siRNAs against ERK2 and JNK1 consistent with the requirement for Exocyst/aPKC in the localised activation of ERK1/2 and JNK1 and the consequent distribution of Paxillin in focal adhesion complexes as opposed to the more static focal adhesion complexes. ERK1, ERK2, and JNK1 were depleted and the effect of their depletion on Paxillin Patches were quantified. Depletion of ERK2 and JNK1 and not ERK1 elicited an increase of Paxillin patches.

## Discussion

It is established here that aPKC isoforms via the Exocyst complex can confer efficient migration through their ability to control the leading edge activation of a distinct subpopulation of MAPKinases, conferring increased speed on the serum-dependent migratory response of cells. This localised process is enabled through the traffic of an aPKC-Exocyst complex to the leading edge of migrating cells. Assembly of this complex is dependent upon Kibra, which appears to act as a scaffold linking aPKC [17],[22] with Sec3 through non-overlapping binding domains. Whilst assembly is not dependent upon aPKC activity, it is promoted by migratory conditions and immuno-isolation of the Exocyst-aPKC complex demonstrates that the associated aPKC is catalytically active. The Exocyst complex is required for aPKC accumulation at the leading edge. Reciprocally, the association of the Exocyst with active aPKC correlates with a requirement for aPKC expression and activity for the traffic of the Exocyst to the leading edge of migrating cells. The Exocyst-Kibra-aPKC complex traffics in a microtubule-dependent fashion to the leading edge of migrating NRK cells and this is a necessary event to promote efficient/directed migration. The control of aPKCs on the Exocyst at the leading edge could be explained by control of microtubule dynamics by aPKC as suggested previously [32]. Consistent with this pattern of behaviour, knock-down of Exocyst subunits or aPKC isoforms, or inhibition of aPKC isoforms (Gö6983, a pan-PKC inhibitor) inhibits migration.

The migratory model itself requires the presence of serum and matrix interactions for migration. For NRK cells, migration occurs on fibronectin, laminin, and collagen with all three displaying a requirement for aPKC for optimum migration. By contrast in RPE1 cells, migration on fibronectin is aPKC-dependent but on laminin or collagen migration is relatively insensitive to knock-down of aPKC. As evident from the NRK cell model here, which is 60% dependent on aPKC, there are multiple modes of migration that display differential dependence on control mechanisms and these vary between cells.

Mechanistically, the aPKC-Exocyst assembly in the NRK cell model confers JNK and ERK activation at the leading edge and furthermore JNK1 (and not JNK2) as well as ERK2 (and not ERK1) inhibition blocks cell migration. JNK's effects appear to be mediated in part through the phosphorylation of Paxillin on serine 178, determining the dynamics of focal adhesions [31],[33]. aPKCs also control the phosphorylation of Paxillin by ERK1/2 on serine 126 (unpublished data). These phosphorylations were described to be important for the turnover of Paxillin at the focal adhesions [27],[31],[33],[34]. Indeed, the knock-down of aPKCζ/ι or Sec5 (or Exo84) causes Paxillin accumulation in large, static focal adhesions. Thus we have mapped a pathway from the assembly of the aPKC-Exocyst complex, through their mutual delivery to the leading edge of migrating cells, the activation there of ERK and JNK, and the consequent phosphorylation of Paxillin, influencing the dynamics of focal adhesion turnover and migration. Video microscopy experiments (Videos S1 and S2) are compatible with the regulation of Paxillin dynamics being under aPKC control. Co-immunoprecipitation experiments showed also an interaction between Sec8 and Paxillin in NRK cells. Paxillin was shown recently as a partner of Sec5 [35]. This interaction between Sec8 and Paxillin increased during NRK cell migration (Figure 6). These data suggest that there is an acute regulation of Paxillin by aPKC.

**Figure 7:**
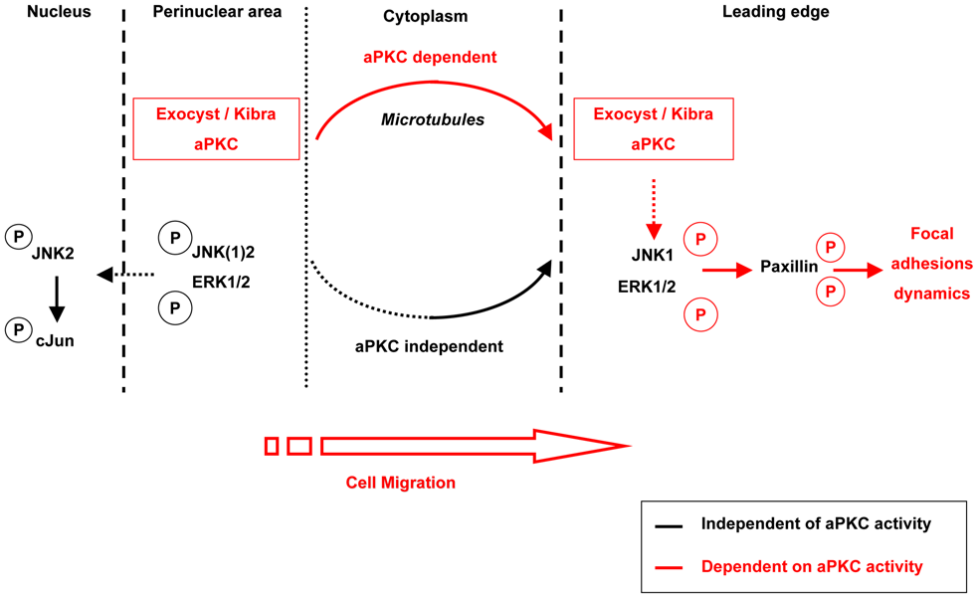
Schematic representation of the aPKC-exocyst complex in cell migration. The Exocyst-Kibra-aPKC complex controls the activation, but not the localisation of JNK1 and ERK1/2 at the leading edge, via a microtubule dependent process. The active JNK1 and ERK1/2 can then phosphorylate the common substrate, Paxillin, determining the stability of focal adhesion dynamics. By contrast, the activation of JNK2 and c-Jun in the nucleus during cell migration is independent of the Exocyst-aPKC pathway.

The action of aPKCs in conferring this promigratory behaviour reflects their role in directing the subcellular localisation of signals. Such spatially resolved behaviour of cellular regulators is increasingly recognised as a critical factor in determining the nature of their output. Here the evidence is for the localised action of the JNK pathway (in particular JNK1) in migration. The depletion of JNK1 decreases the P-JNK staining at the leading edge. Only JNK1 and not JNK2 knock-down inhibits cell migration and Gö6983 inhibits only the phosphorylation of JNK1 and not JNK2 during cell migration. These three observations provide compelling evidence that JNK1 is a key player in the JNK pathway responsible for aPKC-dependent NRK cell migration. Activation of JNK1 at the leading edge, effected through the aPKC-Kibra-Exocyst complex, is necessary for the phosphorylation of Paxillin in this compartment, while the migration-associated, JNK-dependent phosphorylation of nuclear c-jun is immune to aPKC-Kibra^−^-Exocyst function. Conversely it is evident that the activated JNK engaged in c-jun phosphorylation (probably JNK2) is not able to trigger Paxillin phosphorylation at the plasma membrane (Figure 7). The aPKC-dependent plasma membrane activation of both JNK and ERK is driven by delivery of upstream controls and not through the localisation of the JNK or ERK proteins themselves (Figure 7). This lack of effect on ERK and JNK recruitment at the leading edge when the Exocyst-aPKCs is disrupted by various siRNAs shows that a leading edge is preserved; aPKC/Exocyst disruption does not disorganise globally the leading edge, and cells still retain oriented protrusions. Moreover, the distribution of the actin cytoskeleton of migrating cells at the wound edge is retained as described by Guo et al. using siRNA against Exo70 [15]. These MAPKinases along with characteristic cortical actin structures retain their leading edge location independent of aPKC-Kibra-Exocyst action and their activation by phosphorylation. It is implicit that the polarised delivery and/or retention of these MAPKinases at the leading edge is dependent on distinct non-aPKC signal(s).

The upstream signals required for the activation of JNK appears to involve MKK4 and not MKK7 since the former shows sensitivity to inhibition of aPKC (pan-PKC) in its migration-induced activation, while the latter is insensitive. Although the depletion of MKK4 inhibits cell migration consistent with the proposed role for MKK4, so does the depletion of MKK7, preventing distinction to be made between these two JNK kinases. However, it is notable that the morphology of cells depleted of MKK4 (but not of MKK7) is a phenocopy of the cells depleted of aPKC (bigger, more spread cells), consistent with the selective effects of aPKC inhibition on MKK4; cells depleted of MKK7 displayed a stressed appearance after wounding that was quite distinct from the morphological phenotype of MKK4 and aPKC knock-down cells. It is concluded that MKK7, though not activated during migration, is probably required for migration in an aPKC independent pathway.

PKCι can control JNK via Par6 and Rac [36], however the control exerted during cell migration via MEK4 remains under investigation. HGK, a MAPKKKKinase specific for the JNK pathway, was described to interact with the Exocyst complex [37]. So one possibility is that aPKCs could control the interaction of HGK with Exocyst complex. Although the Exocyst has been associated with secretory events, the upstream trigger for activation is not thought to be dependent upon any factor(s) secreted following movement of the Exocyst to the leading edge, since conditioned medium from wounded cells does not rescue wounded cells where the Exocyst subunit Sec5 has been knocked down (Figure S3E, S3F). It would appear that the trigger for activation derives from changes in cell-cell/matrix interactions triggered by removal of cells from the monolayer (wounding).

In conclusion, aPKCs via the Exocyst complex and Kibra are shown to exert a pro-migratory role in NRK cells and do so through the regulated delivery of a signal to the leading edge of migrating cells through kinases upstream of JNK1 and ERK1/2. This regulation of the ERK and JNK pathways via aPKCs allows the phosphorylation of a common substrate, Paxillin (Figure 7), and consequently probably the turnover of Paxillin at the focal adhesion sites. The combined actions of this complex thus integrate the polarised, leading edge delivery of signals required for efficient migration.

## Material and Methods

### Cell Culture and Wound Assay

Normal rat kidney (here denoted NRK, but specifically NRK-49F cells, confirmed by ß-catenin staining [38]; Figure S3A) cells were cultivated in Dulbecco's modified Eagle medium and 10% fetal calf serum under 5% CO_2_ on Falcon plastic dishes. Wounds were inflicted by scratching the cell surface with a plastic pipette tip. Images were recorded using a Zeiss microscope and an Orca ER CCD camera (Hamamatsu). All inhibitor treatments were performed without pre-incubation. Quantification of the speed of individual cells was performed using Metamorph, Tracker, and Mathematica software.

### Immunofluorescence

Cells were fixed in 4% paraformaldehyde (unless stated otherwise), permeabilized in 1% Triton X-100, and mounted using Prolong (Molecular Probes). Primary antibodies were obtained from Stressgen (Sec6), BD Bioscience (Sec8), Cell Signaling (phospho-extracellular signal-regulated kinase 1/2; P-ERK1/2, P-JNK1/2, P-cJun), or Sigma (fluorescein isothiocyanate (FITC)-coupled anti-tubulin). The antibody anti-Exo70 was generously provided by Dr. S. C. Hsu (Department of Cell Biology and Neuroscience, Rutgers University, Piscataway, New Jersey, USA). Secondary antibodies were from Jackson Laboratories and Molecular Probes and were coupled to Cy3 and FITC, respectively. Images were acquired using a confocal laser scanning microscope (LSM510, Carl Zeiss Inc.) equipped with a 63×/1.4 Plan-Apochromat oil immersion objective. Alexa 488 was excited with the 488-nm line of an argon laser, Cy3 was excited with a 543-nm HeNe laser, and Cy5 was excited with a 633-nm HeNe laser. Each image represents a projection or a single section as indicated in the Figure legend.

### Analysis of the Immunofluorescence Data

For experiments involving protein recruitment at the leading edge, at least 70 cells at the wound were counted per experiment according the classification shown in Figure S2. Each figure shows the quantified recruitment of the specified protein at the leading edge from at least three independent experiments.

Where Paxillin spots and Actin stress fibers were quantified, an Array Scan II and the Cellomics analysis program were employed. Each cell was identified by nuclear staining (Dapi) and actin staining (phalloidin). Each area of interest for the analysis (spot or fiber) was delimited and represented as an object. The number of Paxillin spots as well as the total area of stress fibers per cell were measured (Figure S10B).

### Immunoprecipitation and Immunodetection

For whole-cell extracts, cells were lysed directly on plates in hot Laemmli sample buffer. For immunoprecipitation, cells were lysed in 20 mM Tris-HCl (pH 7.4), 100 mM NaCl, 1 mM MgCl_2_, 0.1 mM dithiothreitol, 1% Triton X-100, and 10% glycerol; antibodies were used at the concentrations recommended by suppliers for immunoprecipitation or for immunodetection on membranes. Proteins were visualized on membranes with a chemiluminescent detection system (ECL; Amersham). Quantitation of the immunoprecipitates were performed using Image J. Primary antibodies were obtained from Santa Cruz (PKCζ/ι, JNK1/2, Paxillin), BD Biosciences (PKCι and rSec8), UBI (ERK1/2), cell signaling (P-MKK4, MKK4, P-MKK7, MKK7, P-JNK1/2, P-ERK1/2), or Calbiochem (Phospho-Paxillin [ser178]). The Phospho-SAPK/JNK (Thr183/Tyr185) Blocking Peptide was obtained from Cell Signaling. The different inhibitors (Gö6976, Gö6983, BIM-I, SP600125, and UO126) were obtained from Calbiochem. For “scratch tests,” NRK cells were brought to confluence and scratched orthogonally at least 20 times with a p20-200 yellow tip. Cells were either further incubated for 3 h or harvested immediately. Preparations of cell extracts and co-immunoprecipitation were performed according to published procedures [16]. For detection of the activation of ERK1/2, JNK1/2, c-Jun, and MKK4, cells were scratched, allowed to migrate for different times (as indicated), and then harvested in Laemmli sample buffer, boiled, and processed for Western blotting. For direct comparison all cells were maintained in contact with the inhibitors for a 3 or 6 h period as indicated in the text or figure legends.

### Two Hybrid Screen

Full-length human Sec3 was cloned into pB27, derived from the original pBTM116 plasmid [39], and used as bait to screen a random-primed human placenta cDNA library constructed in pP6 [40]. A total of 120 million clones (12-fold library coverage) were screened using a mating approach with L40ΔGal4 (mata) and Y187 (matα) yeast strains [41]. His+ colonies were selected on medium lacking tryptophan, leucine, and histidine, supplemented with 2mM 3-aminotriazole to reduce bait autoactivation. Prey fragments of the positive clones were amplified by PCR and sequenced at their 5′ and 3′ junctions on a PE3700 Sequencer. The resulting sequences were used to identify the corresponding interactors in the GenBank database (NCBI) using a fully automated procedure.

### siRNAs and Plasmid Constructs

siRNAs were transfected at 10 nM with Hyperfect (Qiagen) according to the recommendations of the manufacturer. siRNA were ordered from Dharmacon (rPKCζ-1) or Proligo (the others). Target sequences were:

GCAAGCUGCUUGUCCAUAAdTdT(rPKCζ-1),

GCAAACUGCUGGUUCAUAAdTdT (rPKCι-1),

GAAGAAAGAGCUCGUCAAUdTdT (rPKCι-2),

GCAGUGAGGUUCGAGAUAUdTdT (rPKCι-3),

GAACGAUGGUGUAGACC U UdTdT (rPKCζ-2).

Sequences for Sec5 were described previously [16]. The sequence for Exo84 is UGGGCAUGUUCGUGGAUGCdTdT.

A smart-pool untargeted plus from Dharmacon was used to deplete rat Kibra and independent siRNAs against rKibra AGGAGAUCUACCAGGUGAAdTdT (Kibra-330), AGCACGACUACAGUUCAAdTdT (rKibra-1), and CCACUCACCUUUGCUGACUdTdT (rKibra-2) were also employed. Myc-PKCζ and PKCι constructs were described previously [42]. Myc-Kibra and Flag-Kibra constructs were described previously [17],[22].

The siRNA for the JNK and ERK pathways were obtained from Qiagen; siJNK1 (SI03083185 and SI03105802) and a smart pool from Dharmacon was also used to deplete rat JNK1, siJNK2 (SI01906310 and SI02723588), siERK1 (SI01300593 and SI01906163), siERK2 (SI02672117 and SI02692326), siMKK4 (SI01533791 and SI01533798), and siMKK7 (SI04404680 and SI04404687).

## Supporting Information

Figure S1 Controls for the recruitment of Exo70 and PKCι at the leading edge. (A–B) Confluent monolayers of NRK cells were wounded (top panels) or cultivated at 70% confluence (lower panels). Cells were fixed 3 h after wounding with paraformaldhehyde, permeabilized with 1% Triton×100, and then immunostained for Exo70, PKCι, and a cytoplasmic tracker (Molecular Probe) as indicated in the individual panels. Each image is a projection of confocal slices. Only in migrating conditions (red arrows), Exo70 and PKCι are recruited at the edge of the cells. There is no recruitment of Exo 70 or of PKCι in non migrating cells (grey arrows).
(7.03 MB TIF)

Figure S2 Quantification of the recruitment at the leading edge of Exo70 and PKCι after depletion of aPKC or a component of the Exocyst (Sec5 or Exo84). (A) Schematic representation illustrating how the cells at the leading edge were quantified. (−) no recruitment at the leading edge, (+) partial recruitment at the leading edge, (++) total recruitment at the leading edge. (B) Quantification of PKCι at the leading edge. (C) Quantification of Exo70 at the leading edge.
(1.32 MB TIF)

Figure S3 The influence of aPKC on the Exocyst and migration is independent of CDC42 and does not reflect a general secretory or membrane traffic effect. (A) A monolayer of NRK cells was fixed with paraformaldhehyde and β-Catenin and Paxillin detected by immunofluorescence. (B) Monolayers of NRK cells were wounded 60 h post-transfection with the indicated siRNAs (10 nM). (C) Monolayers of NRK cells were wounded and stained for Sec6 and β-COP. Monolayers of NRK cells were wounded 60 h post-transfection with the indicated siRNAs (10 nM). Cells were fixed with methanol 60 h after wounding and stained for Sec6 and beta-COP. The images are projections of confocal sections. (D) Monolayers of NRK cells overexpressing dominant negative Myc-CDC42 were wounded and stained for Exo70 and Myc. Cells were fixed with paraformaldhehyde 6 h after wounding and Exo70 detected by immunofluorescence. (E) Quantitation of migration speed and the effects of Sec5 knock-down with or without different conditioned media are summarized in the panel. (F) Controls illustrating Sec5 depletion relating to Panel E. NRK cells were transfected with the indicated siRNA, and after 72 h, whole-cell extracts were analyzed by immunoblotting.
(9.34 MB TIF)

Figure S4 Interaction between the Exocyst and PKCζ and PKCι via Kibra is independent of aPKC activity, but aPKC activity is required for ERK1/2 activation in migrating NRK cells. (A,B) Protein extracts from NRK cells over-expressing PKCι (A) or a myc tag PKCζ construct (B) were subjected to immunoprecipitation with an anti-Sec8 antibody, and the immunoprecipitates were analyzed for the presence of PKCι or mycPKCζ by Western blotting. (C) Protein extracts from NRK cells were subjected to immunoprecipitation with an anti-Sec8 antibody in the presence or absence of the aPKC inhibitor Gö6983. The immunoprecipitates were analyzed for the presence of endogenous PKCζ/ι and PKCι. (D) Monolayers of confluent NRK cells were extensively scratched to maximize the number of “free edges” where cell-cell contacts were released. As indicated NRK cells were pre-treated with the c/n/aPKC inhibitor (Gö6983 at 1 or 5µM) for a total of 6 h. Extracts were prepared at the times indicated, and Phospho-JNK1/2, phospho-ERK1/2, total JNK1/2, total ERK1/2, phospho-p38, Sec8, Sec5, aPKC, and PKCι were detected by Western blotting. (E) NRK cells were treated with PKC inhibitors (Gö6976 and Gö6983 at 5 µM) for a total of 6 h. Cell extracts were prepared at the times indicated and Phospho-ERK1/2 and total ERK1/2 were detected.
(0.99 MB TIF)

Figure S5 Depletion of Kibra or Exo84 disrupts the local phosphorylation of JNK and ERK at the leading edge; depletion of Sec5 or aPKC does not modify the plasma membrane delivery of ERK1/2, JNK1/2 proteins, or the phosphorylation of nuclear cJun during cell migration. (A) Monolayers of NRK cells were wounded 60 h post-transfection with the indicated siRNAs (10 nM). Cells were fixed 6 h after wounding with paraformaldhehyde, permeabilized with 1% Triton×100, and then immunostained for P-JNK, P-ERK, and actin (Phalloidin) as indicated in the individual panels. Each image is a projection of confocal slices. (B) Monolayers of NRK cells were wounded. Cells were treated for the 3 h of migration with UO1206 (20 µM). Cells were then fixed with paraformaldhehyde, and P-ERK1/2 and actin (Phalloidin) were detected with specific antibodies. (C,D,E,F) Monolayers of NRK cells were wounded 60 h post-transfection with the indicated siRNAs (10 nM). (C) Cells were fixed with paraformaldhehyde 6 h after wounding and JNK1/2 detected by immunofluorescence. (D) Cells were fixed with methanol 6 h after wounding and ERK1/2 was detected by immunofluorescence. (E) Cells were fixed with paraformaldhehyde 6 h after wounding and P-cJun detected by immunofluorescence. (F) Cells were fixed with methanol 6 h after wounding and ERK1/2 and P-ERK1/2 were detected by immunofluorescence.
(4.96 MB TIF)

Figure S6 Interaction between Sec8, aPKC, and ectopic Kibra; localization of Kibra in migrating cells. (A) Protein extracts from NRK cells over-expressing Flag-Kibra were subjected to immunoprecipitation with an anti-Flag antibody and the immunoprecipitates were analyzed for the presence of aPKC and Sec8 by Western blotting. (B) A monolayer of confluent NRK cells over-expressing Myc-Kibra was extensively scratched to maximize the number of “free edges” where cell-cell contacts were released. Sec8 was immunoprecipitated and Myc Kibra was detected by Western blot. (C) Monolayers of NRK cells were wounded 48 h post-transfection with a Flag-Kibra construct. Cells were fixed with paraformaldhehyde 3 h after wounding and Flag-Kibra and aPKC were detected by immunofluorescence. (D) Control of the experiment Figure 3H. NRK cells were transfected with a siRNA against Kibra or an siRNA control as indicated. After 72 h of transfection, the 1% (v/v) Triton×100 soluble fractions were analyzed by immunoblotting with the indicated antibodies.
(4.64 MB TIF)

Figure S7 Controls for the depletion by siRNAs of aPKCs, the Exocyst, ERK, MKK and JNK proteins, and immunostaining of P-ERK and P-JNK. (A, B, C, D, E) siRNA specificity and efficiency in NRK cells. NRK cells were transfected with the indicated siRNAs, and after 72 h whole-cell extracts were analyzed by immunoblotting with the indicated antibodies. (F) Monolayers of NRK cells were wounded and were fixed with paraformaldhehyde 6 h after wounding. P-JNK1/2 and P-ERK1/2 were detected by immunofluorescence. The antibodies were preincubated with or without a phospho-peptide competitor of P-JNK1/2 (0.1 µg/ml). (G) Monolayers of NRK cells were wounded 60 h post-transfection with the indicated siRNAs (20 nM). Cells were fixed with paraformaldhehyde 6 h after wounding, and P-JNK1/2 and P-ERK1/2 were detected by immunofluorescence. (H) NRK cells were treated with the MEK inhibitor UO126 (10 µM) or with the JNK inhibitor SP600125 (20 µM). P-cJun (ser63), P-Paxillin (ser178), and total cJun were detected. (I) After 6 h of treatment with SP600125 at 20 µM to inhibit JNK activity, confluent monolayers of NRK cells were wounded. The healing was followed for a further 12 h. T0 and T12 refer respectively to 0 h and 12 h post-wounding. The panels shown are representative at least of three independent experiments.
(4.40 MB TIF)

Figure S8 Quantification of the recruitment to the leading edge of P-ERK, Total ERK, P-JNK, and total JNK after depletion of aPKC or one component of the Exocyst (Sec5 or Exo84). (A) Schematic representation illustrating how the cells at the leading edge were quantified. (−) no recruitment at the leading edge, (+) partial recruitment at the leading edge, (++) total recruitment at the leading edge. (B) Quantification of P-ERK and total ERK at the leading edge. (C) Quantification of P-JNK and total JNK at the leading edge.
(1.73 MB TIF)

Figure S9 Quantification of number of spots of Paxillin per cell and the area of stress fibers after depletion of aPKC or one component of the Exocyst (Sec5 or Exo84). (A,B) In a 96 well plate, NRK cells were transfected with siRNA against aPKCs, Sec5, Exo84, or an siRNA control as indicated. After 48 h of transfection, cells were split and re-plated on fibronectin. The day after (72 h post-transfection), cells were fixed in paraformaldehyde 4% and stained for paxillin and actin (phalloidoin). The staining was analysed by Array Scan II and a Cellomics analysis program. (A) Quantification of the number of spots of Paxillin per cell. (B) Quantification of the area of stress fibers per cell.
(1.24 MB TIF)

Figure S10 Quantification of the number of spots of Paxillin per cell and the area of stress fibers after depletion of JNK1, ERK1, or ERK2 (as indicated). (A) In a 96 well plate, NRK cells were transfected with a siRNA against JNK1, ERK1, ERK2, or a siRNA control as indicated. After 48 h of transfection, cells were split and re-plated on fibronectin. The day after (72 h post-transfection), cells were fixed in paraformaldehyde 4% and stained for paxillin and actin (phalloidoin). The staining was analysed by Array Scan II and a Cellomics analysis program. The panel indicates the quantification of the number of spots of Paxillin per cell. (B) Illustration of the quantification of Paxillin and actin (phalloidin stained) structures. In a 96 well plate, NRK cells were transfected with siRNAs. After 48 h, cells were split and re-plated on fibronectin. The following day (72 h post-transfection), cells were fixed in paraformaldehyde (4%) and stained for paxillin, nuclei (dapi), and actin (phalloidoin). The staining was analysed by Array Scan II and a Cellomics analysis program: Each cell was identified by nuclear staining and actin staining. The area of interest for the analysis (spot or fiber) was delimited and represented as an object (Masks). The number of Paxillin spots as well as the total area of stress fibers per cell were measured.
(4.38 MB TIF)

Video S1 Dynamics of GFP-Paxillin during cell migration (control). Monolayers of NRK cells were wounded 60 h post-transfection with siRNA (20 nM) against Luciferase and 48 h post-transfection with GFP-Paxillin. A TIRF microscope was used, and images were taken every minute during 1 h.
(0.40 MB MOV)

Video S2 Dynamics of GFP-Paxillin during cell migration in absence of aPKC. Monolayers of NRK cells were wounded 60 h post-transfection with siRNA (20 nM) against PKCζ/ι and 48 h post-transfection with GFP-Paxillin. A TIRF microscope was used, and images were taken every minute during 1 h.
(0.37 MB MOV)

## Abbreviations

APC: adenomatous polyposis coli
aPKC: atypical PKC
β-COP: beta Coat Protein Complex
ERK: extracellular regulated kinase
JNK: c-jun N-terminal kinase
MAPK: mitogen activated protein kinase
MEK: MAPK or ERK kinase
MKK: MAPK kinase kinase PKC, Protein Kinase C
RNAi: RNA interference
