# Supplementary figures and images for: An aPKC-Exocyst Complex Controls Paxillin Phosphorylation and Migration through Localised JNK1 Activation
Source: PLoS Biol. 2009 Nov 3;7(11):e1000235. doi: 10.1371/journal.pbio.1000235 (PMC2762617; doi:10.1371/journal.pbio.1000235)

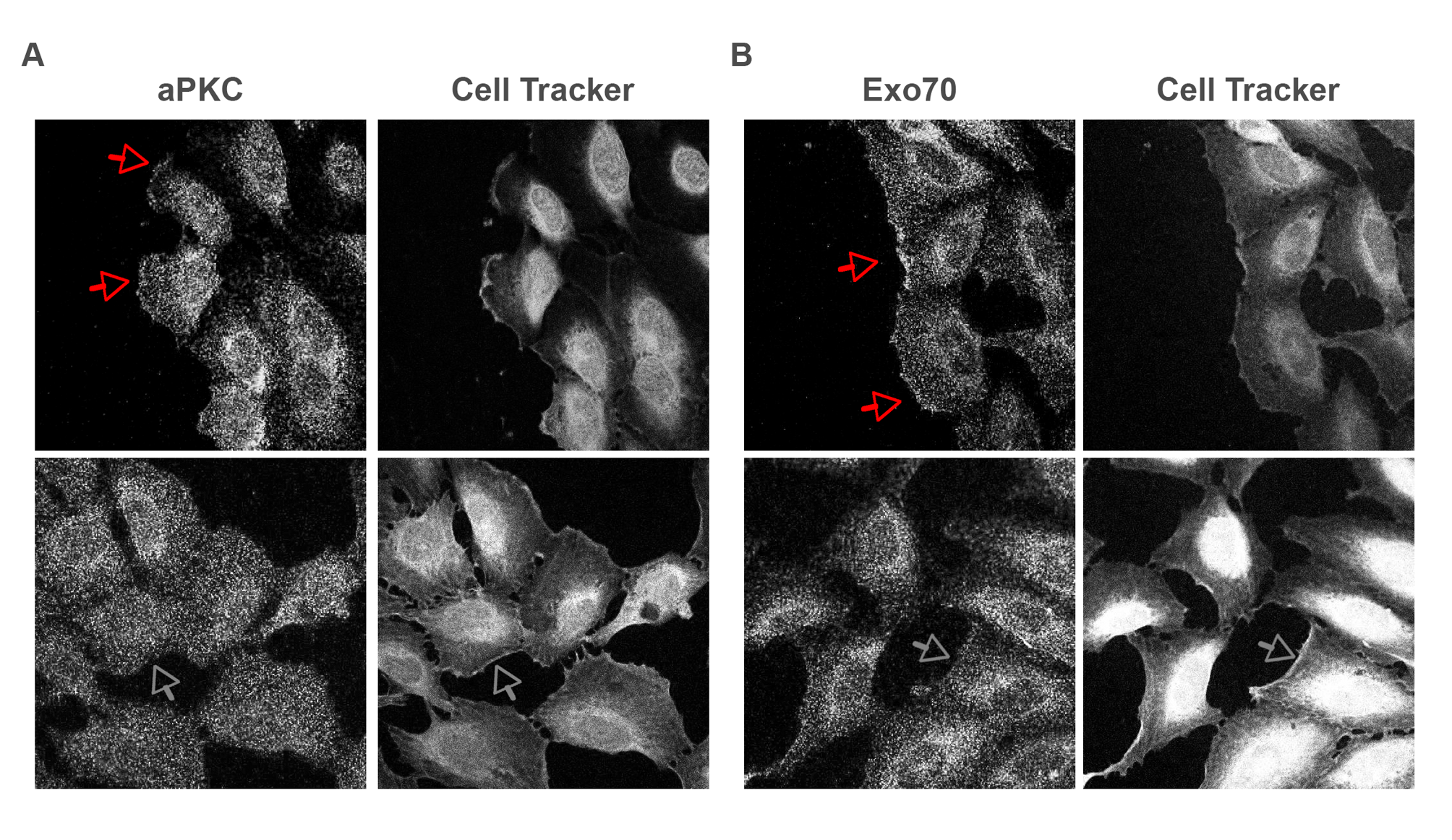

Supplement: Figure S1 — Controls for the recruitment of Exo70 and PKCι at the leading edge. (A–B) Confluent monolayers of NRK cells were wounded (top panels) or cultivated at 70% confluence (lower panels). Cells were fixed 3 h after wounding with paraformaldhehyde, permeabilized with 1% Triton×100, and then immunostained for Exo70, PKCι, and a cytoplasmic tracker (Molecular Probe) as indicated in the individual panels. Each image is a projection of confocal slices. Only in migrating conditions (red arrows), Exo70 and PKCι are recruited at the edge of the cells. There is no recruitment of Exo 70 or of PKCι in non migrating cells (grey arrows). (7.03 MB TIF) [file pbio.1000235.s001.tif]

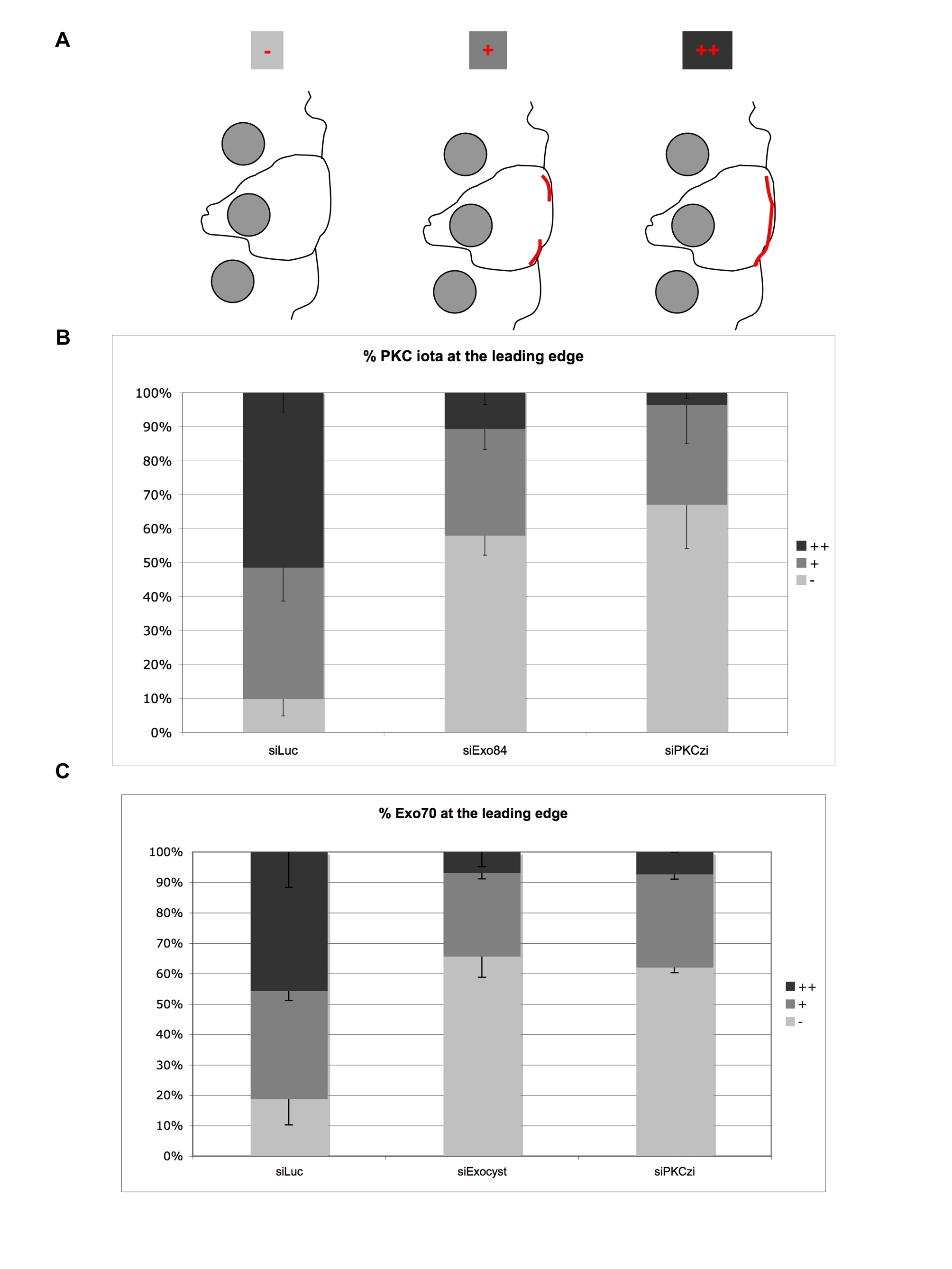

Supplement: Figure S2 — Quantification of the recruitment at the leading edge of Exo70 and PKCι after depletion of aPKC or a component of the Exocyst (Sec5 or Exo84). (A) Schematic representation illustrating how the cells at the leading edge were quantified. (−) no recruitment at the leading edge, (+) partial recruitment at the leading edge, (++) total recruitment at the leading edge. (B) Quantification of PKCι at the leading edge. (C) Quantification of Exo70 at the leading edge. (1.32 MB TIF) [file pbio.1000235.s002.tif]

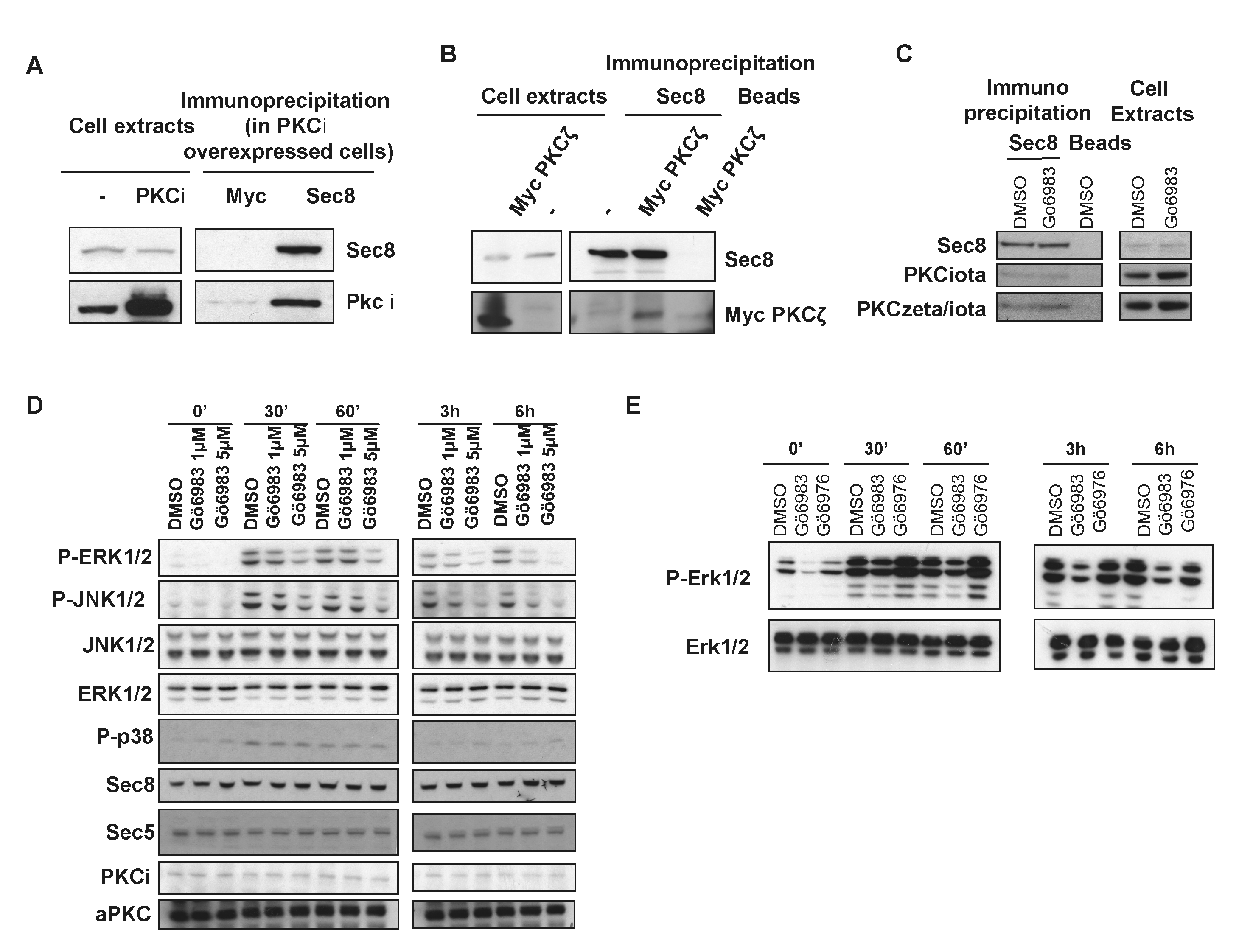

Supplement: Figure S4 — Interaction between the Exocyst and PKCζ and PKCι via Kibra is independent of aPKC activity, but aPKC activity is required for ERK1/2 activation in migrating NRK cells. (A,B) Protein extracts from NRK cells over-expressing PKCι (A) or a myc tag PKCζ construct (B) were subjected to immunoprecipitation with an anti-Sec8 antibody, and the immunoprecipitates were analyzed for the presence of PKCι or mycPKCζ by Western blotting. (C) Protein extracts from NRK cells were subjected to immunoprecipitation with an anti-Sec8 antibody in the presence or absence of the aPKC inhibitor Gö6983. The immunoprecipitates were analyzed for the presence of endogenous PKCζ/ι and PKCι. (D) Monolayers of confluent NRK cells were extensively scratched to maximize the number of “free edges” where cell-cell contacts were released. As indicated NRK cells were pre-treated with the c/n/aPKC inhibitor (Gö6983 at 1 or 5µM) for a total of 6 h. Extracts were prepared at the times indicated, and Phospho-JNK1/2, phospho-ERK1/2, total JNK1/2, total ERK1/2, phospho-p38, Sec8, Sec5, aPKC, and PKCι were detected by Western blotting. (E) NRK cells were treated with PKC inhibitors (Gö6976 and Gö6983 at 5 µM) for a total of 6 h. Cell extracts were prepared at the times indicated and Phospho-ERK1/2 and total ERK1/2 were detected. (0.99 MB TIF) [file pbio.1000235.s004.tif]

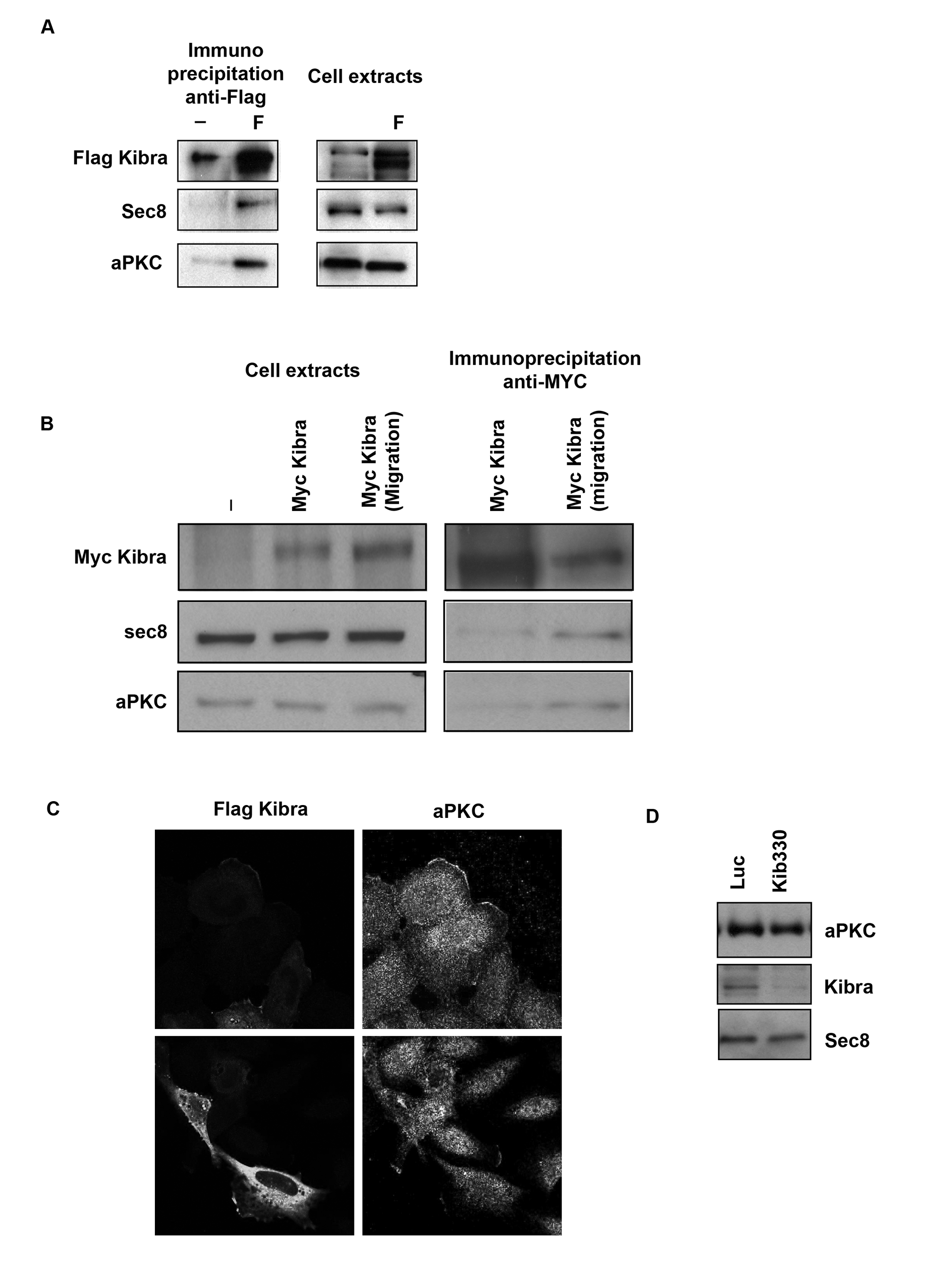

Supplement: Figure S5 — Depletion of Kibra or Exo84 disrupts the local phosphorylation of JNK and ERK at the leading edge; depletion of Sec5 or aPKC does not modify the plasma membrane delivery of ERK1/2, JNK1/2 proteins, or the phosphorylation of nuclear cJun during cell migration. (A) Monolayers of NRK cells were wounded 60 h post-transfection with the indicated siRNAs (10 nM). Cells were fixed 6 h after wounding with paraformaldhehyde, permeabilized with 1% Triton×100, and then immunostained for P-JNK, P-ERK, and actin (Phalloidin) as indicated in the individual panels. Each image is a projection of confocal slices. (B) Monolayers of NRK cells were wounded. Cells were treated for the 3 h of migration with UO1206 (20 µM). Cells were then fixed with paraformaldhehyde, and P-ERK1/2 and actin (Phalloidin) were detected with specific antibodies. (C,D,E,F) Monolayers of NRK cells were wounded 60 h post-transfection with the indicated siRNAs (10 nM). (C) Cells were fixed with paraformaldhehyde 6 h after wounding and JNK1/2 detected by immunofluorescence. (D) Cells were fixed with methanol 6 h after wounding and ERK1/2 was detected by immunofluorescence. (E) Cells were fixed with paraformaldhehyde 6 h after wounding and P-cJun detected by immunofluorescence. (F) Cells were fixed with methanol 6 h after wounding and ERK1/2 and P-ERK1/2 were detected by immunofluorescence. (4.96 MB TIF) [file pbio.1000235.s005.tif]

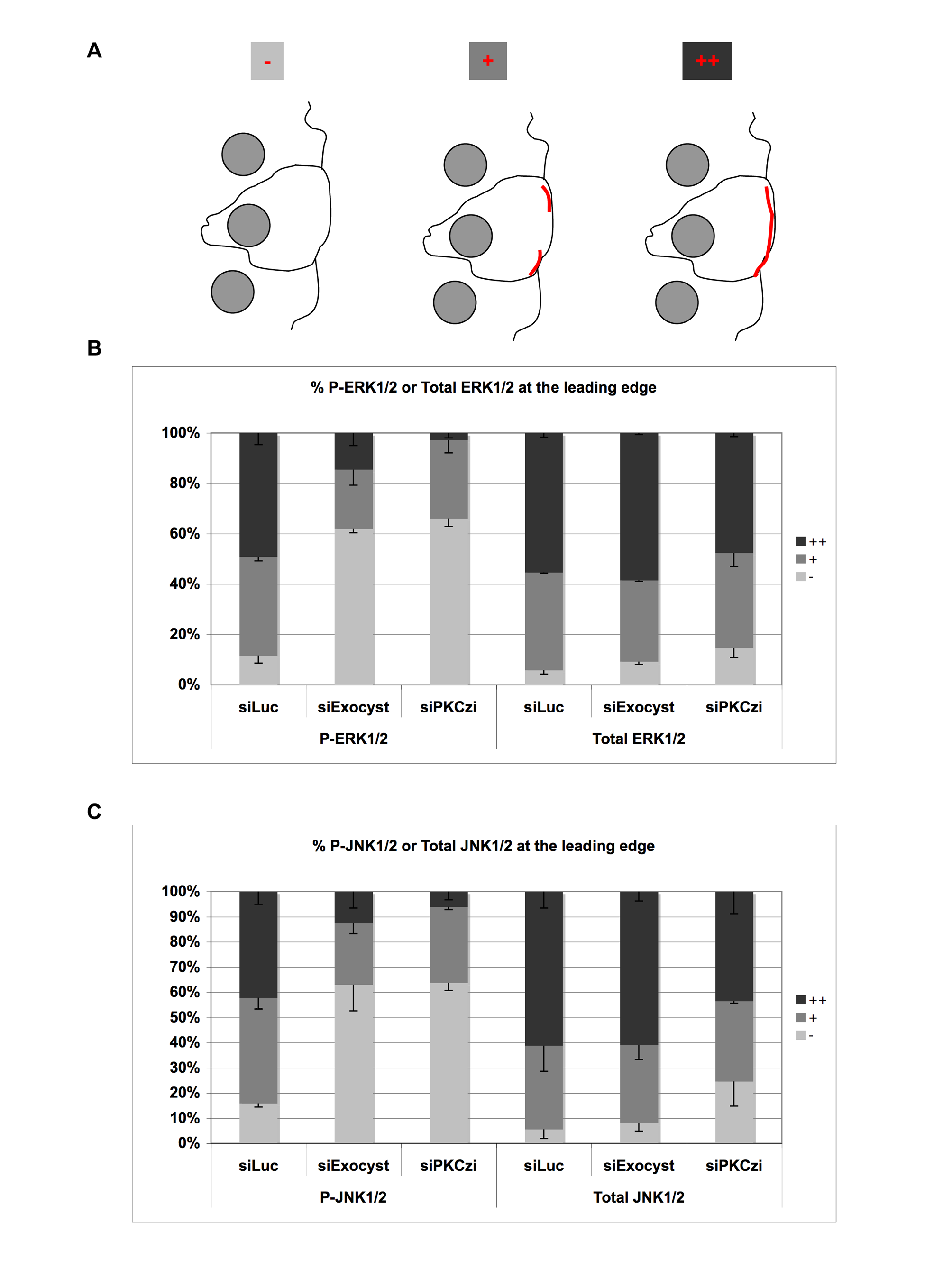

Supplement: Figure S8 — Quantification of the recruitment to the leading edge of P-ERK, Total ERK, P-JNK, and total JNK after depletion of aPKC or one component of the Exocyst (Sec5 or Exo84). (A) Schematic representation illustrating how the cells at the leading edge were quantified. (−) no recruitment at the leading edge, (+) partial recruitment at the leading edge, (++) total recruitment at the leading edge. (B) Quantification of P-ERK and total ERK at the leading edge. (C) Quantification of P-JNK and total JNK at the leading edge. (1.73 MB TIF) [file pbio.1000235.s008.tif]

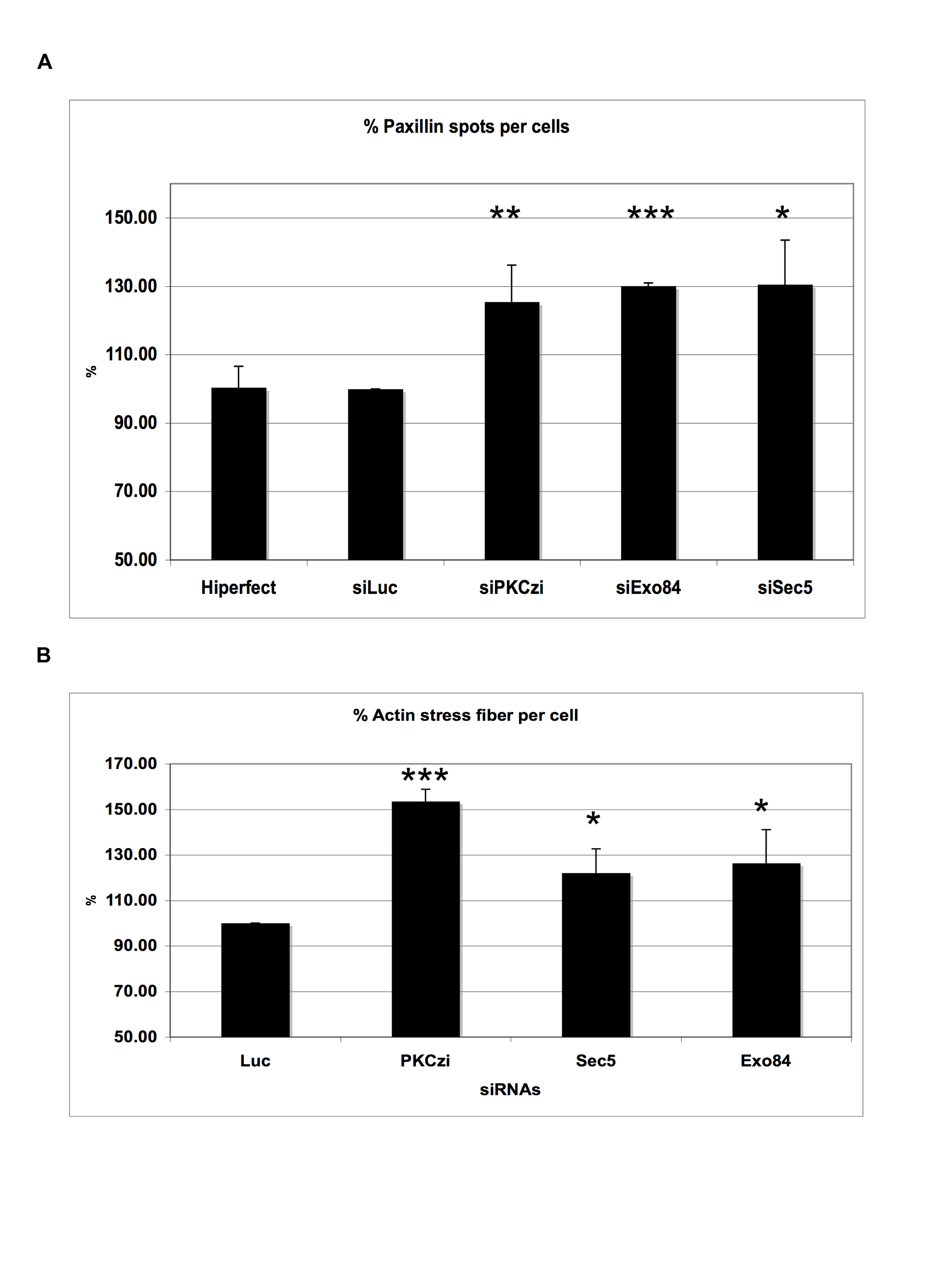

Supplement: Figure S9 — Quantification of number of spots of Paxillin per cell and the area of stress fibers after depletion of aPKC or one component of the Exocyst (Sec5 or Exo84). (A,B) In a 96 well plate, NRK cells were transfected with siRNA against aPKCs, Sec5, Exo84, or an siRNA control as indicated. After 48 h of transfection, cells were split and re-plated on fibronectin. The day after (72 h post-transfection), cells were fixed in paraformaldehyde 4% and stained for paxillin and actin (phalloidoin). The staining was analysed by Array Scan II and a Cellomics analysis program. (A) Quantification of the number of spots of Paxillin per cell. (B) Quantification of the area of stress fibers per cell. (1.24 MB TIF) [file pbio.1000235.s009.tif]

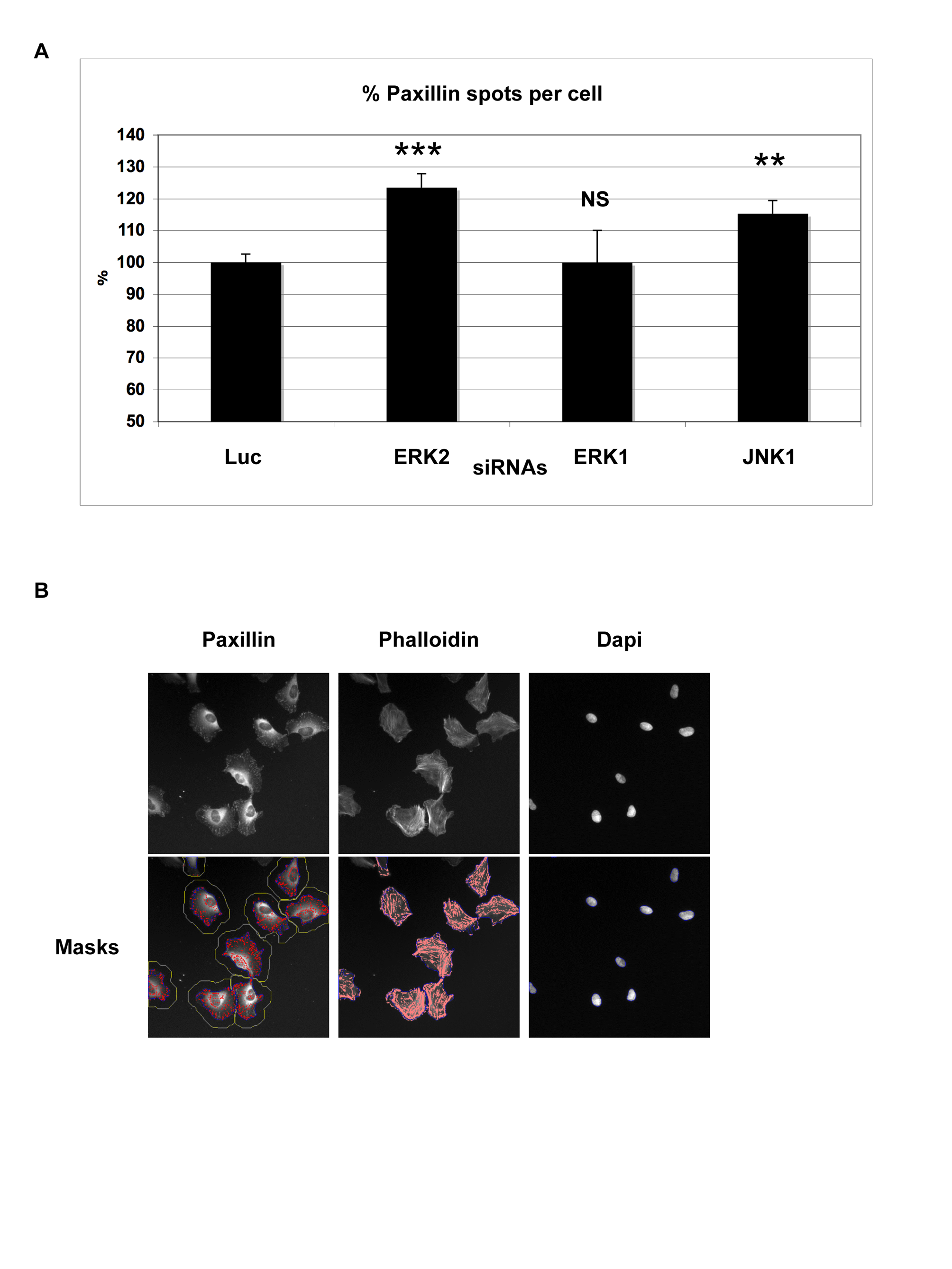

Supplement: Figure S10 — Quantification of the number of spots of Paxillin per cell and the area of stress fibers after depletion of JNK1, ERK1, or ERK2 (as indicated). (A) In a 96 well plate, NRK cells were transfected with a siRNA against JNK1, ERK1, ERK2, or a siRNA control as indicated. After 48 h of transfection, cells were split and re-plated on fibronectin. The day after (72 h post-transfection), cells were fixed in paraformaldehyde 4% and stained for paxillin and actin (phalloidoin). The staining was analysed by Array Scan II and a Cellomics analysis program. The panel indicates the quantification of the number of spots of Paxillin per cell. (B) Illustration of the quantification of Paxillin and actin (phalloidin stained) structures. In a 96 well plate, NRK cells were transfected with siRNAs. After 48 h, cells were split and re-plated on fibronectin. The following day (72 h post-transfection), cells were fixed in paraformaldehyde (4%) and stained for paxillin, nuclei (dapi), and actin (phalloidoin). The staining was analysed by Array Scan II and a Cellomics analysis program: Each cell was identified by nuclear staining and actin staining. The area of interest for the analysis (spot or fiber) was delimited and represented as an object (Masks). The number of Paxillin spots as well as the total area of stress fibers per cell were measured. (4.38 MB TIF) [file pbio.1000235.s010.tif]
